# Quantitative Trait Loci Affecting Atherosclerosis at the Aortic Root Identified in an Intercross between DBA2J and 129S6 Apolipoprotein E-Null Mice

**DOI:** 10.1371/journal.pone.0088274

**Published:** 2014-02-20

**Authors:** Yukako Kayashima, Hirofumi Tomita, Svetlana Zhilicheva, Shinja Kim, Hyung-Suk Kim, Brian J. Bennett, Nobuyo Maeda

**Affiliations:** 1 Department of Pathology and Laboratory Medicine, University of North Carolina at Chapel Hill, Chapel Hill, North Carolina, United States of America; 2 Department of Genetics, University of North Carolina at Chapel Hill, Chapel Hill, North Carolina, United States of America; Westfaelische Wilhelms Universitaet, Germany

## Abstract

Apolipoprotein E-null mice on a DBA/2J genetic background (DBA-apoE) are highly susceptible to atherosclerosis in the aortic root area compared with those on a 129S6 background (129-apoE). To explore atherosclerosis-responsible genetic regions, we performed a quantitative trait locus (QTL) analysis using 172 male and 137 female F2 derived from an intercross between DBA-apoE and 129-apoE mice. A genome-wide scan identified two significant QTL for the size of lesions at the root: one is *Ath44* on Chromosome (Chr) 1 at 158 Mb, and the other *Ath45* on Chr 2 at 162 Mb. *Ath44* co-localizes with but appears to be independent of a previously reported QTL, *Ath1*, while *Ath45* is a novel QTL. DBA alleles of both *Ath44* and *Ath45* confer atherosclerosis-susceptibility. In addition, a QTL on Chr 14 at 73 Mb was found significant only in males, and 129 allele conferring susceptibility. Further analysis detected female-specific interactions between a second QTL on Chr 1 at 73 Mb and a QTL on Chr 3 at 21 Mb, and between Chr 7 at 84 Mb and Chr 12 at 77 Mb. These loci for the root atherosclerosis were independent of QTLs for plasma total cholesterol and QTLs for triglycerides, but a QTL for HDL (Chr 1 at 126 Mb) overlapped with the *Ath44*. Notably, haplotype analysis among 129S6, DBA/2J and C57BL/6 genomes and their gene expression data narrowed the candidate regions for *Ath44* and *Ath45* to less than 5 Mb intervals where multiple genome wide associations with cardiovascular phenotypes have also been reported in humans. SNPs in or near *Fmo3*, *Sele* and *Selp* for *Ath44*, and *Lbp* and *Pkig* for *Ath45* were suggested for further investigation as potential candidates underlying the atherosclerosis susceptibility.

## Introduction

Atherosclerosis is a major cause of myocardial infarction and stroke. Risk factors for atherosclerosis include smoking, obesity, diabetes, hypertension and high plasma cholesterol level, suggesting that formation and progression of atherosclerotic plaques are influenced by complex interactions between multiple genetic and environmental factors. Recent advances in genome-wide association studies (GWAS) of human populations have led to the identification of a number of loci associated with atherosclerosis [1]. However, except for those such as LDLR and apoE that affect plasma cholesterol levels, roles of other genetic factors that influence early pathogenesis of atherosclerosis are not fully understood [2].

Inbred strains of mice show a huge variance in atherosclerotic susceptibility; in apoE-null mice on the different genetic backgrounds, for example, the lesion size in the aortic root areas increases in the order of DBA/2J>C57BL/6J >129> AKR/J>BALB/cJ>C3H/HeJ [3], [4]. By utilizing the strain-specific variations in atherosclerosis susceptibility, quantitative trait locus (QTL) analyses on different sets of intercross have identified more than 40 chromosomal loci that affect atherosclerotic plaque sizes [3], [5], [6], [7], [8]. Genetic studies in mice primarily focus on the very early stages of atherosclerosis, such as plaque initiation and growth at the aortic root, which complement GWAS where loci are primarily associated with advanced atherosclerosis or clinical vascular events. Thus, although most of the regions described in the mouse QTL mapping studies do not contain genes identified in human GWAS, several strong candidate genes have been proposed. These include *Tnfsf4* on Chr 1 [9], *Rcn2* on Chr 9 [10], *Tnfaip3* on Chr 10 [11], *Raet1e* on Chr 10 [12], and *Adam17* on Chr 12 [13]. Studies have also demonstrated that the bioinformatics approaches such as combined-cross analysis and haplotype analysis help reduce the number of the candidate genes [14]. Most QTL studies in mice have utilized the C57BL/6J strain due to their high susceptibility to atherosclerosis. However, systematic QTL analyses where strains other than C57BL/6J are utilized will be highly informative and can reveal new QTL and do provide an important data source for bioinformatics analyses. Furthermore, mouse studies compensate for human studies by controlling environmental factors and focusing on earlier stages of the progression of atherosclerosis. Recent expansion in genome information available for inbred strains of mice also provides a highly promising approach accelerating this process through genome comparison.

Previously, we have shown that apolipoprotein E-null mice on a C57BL/6J and a 129S6 genetic background (B6-apoE and 129-apoE) develop atherosclerosis differently in the aortic arch and the aortic root area: in the arches, 129-apoE mice form larger plaques than B6-apoE mice, whereas plaques in the roots are smaller in 129-apoE than in B6-apoE [4]. QTL analysis on the F2 generations of 129-apoE and B6-apoE cross identified significant QTL for the arch lesion on Chr 1 and for the root lesion on Chr 9, demonstrating that different genetic factors are likely contributing to the strain- and location-specific differences of plaque development [15]. In the present study, we carried out QTL analysis in an additional intercross between apolipoprotein E-null mice on a DBA/2J and those on a 129S6 genetic background, and identified two significant QTL for the root lesion; *Ath44* on Chr 1 which overlaps with but appears to be independent from the previously identified *Ath1* [16], and a novel locus *Ath45* on Chr 2. We narrowed the candidate regions of each QTL using bioinformatics information.

## Results

### Atherosclerotic Plaque Size and Plasma Lipids in the Parental, F1, and F2 Mice

**Figure 1:**
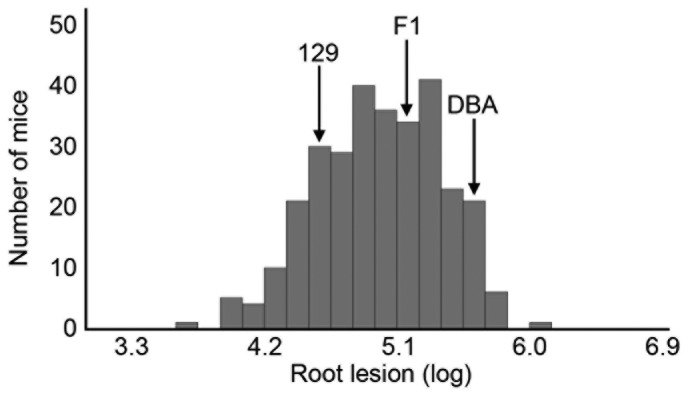
Distributions of atherosclerotic plaque sizes at the root area. Histograms of log-transformed atherosclerotic plaque sizes at the aortic root in 309 F2 mice derived from 129-apoE and DBA-apoE mice. Arrows represent the positions of average root plaque sizes for parental 129-apoE, DBA-apoE and F1 mice.

Atherosclerotic plaque sizes in the aortic root area and plasma lipids were measured in parental 129-apoE and DBA-apoE, F1, and F2 mice (Table 1). Plaque sizes in the aortic root of male mice were approximately 9 times larger in parental DBA-apoE than in 129-apoE mice. Similarly, root plaque sizes in female mice were approximately 14 times larger in DBA-apoE than in 129-apoE mice. The striking differences in the root plaque sizes between DBA-apoE and 129-apoE mice are consistent with a previous report [3]. Neither DBA-apoE nor 129-apoE mice exhibited statistically significant sex-dependent differences. In the F1 and F2 mice, the plaque size distributions were intermediate between those in the parental strains. Since root lesion sizes and plasma lipids were not normally distributed, log-transformed values were used for QTL analysis (Table S1, Figure 1 and Figure S1).

**Table 1:** Atherosclerotic plaque sizes at the aortic root area and plasma lipid levels in the parental, F1, and F2 mice.

		129-apoE (n)	DBA-apoE (n)	F1 (n)	F2 (n)
Root lesion, 10^4^ μm^2^	Male	5.1±2.8 (15)	43.7±8.5 (13)^c^	22.2±10.1 (11)	12.1±11.8 (190)
	Female	3.6±1.5 (17)	51.9±11.3 (13)^b^	11.6±4.3 (9)	17.7±14.7 (149)
T-Chol, mg/dL	Male	898±104 (15)	671±124 (18)^b^	867±209 (14)	891±200 (203)
	Female	628±168 (18)	665±205 (18)	673±115 (9)	740±185 (152)
HDL-C, mg/dL	Male	71.3±16.1 (15)	61.4±25.6 (18)	81.1±44.0 (11)	83.6±33.2 (203)
	Female	80.4±36.1(18)	57.8±13.9 (18)^a^	72.4±18.0 (9)	77.1±33.4 (152)
TG, mg/dL	Male	106±40 (15)	186±92 (18)^b^	256±106 (14)	247±115 (203)
	Female	68.3±30 (18)	166±82 (18)^b^	174±45 (9)	149±67 (152)

Data are shown as the mean ± SD.

a p<0.05,

b p<0.01,

c p<0.001 vs. 129-apoE mice within each sex.

DBA-apoE mice have lower plasma HDL-cholesterol and higher triglyceride levels than 129-apoE mice. Plasma total cholesterol levels were lower in DBA-apoE than in 129-apoE mice in males only (Table 1). While increased plasma lipid levels are potentially atherogenic, there is only a very small association between the lipid levels and lesion sizes in the F2 population (Figure S2). Approximately 4% of the variation in the lesion size can be attributed to the total cholesterol (R^2^ = 0.04, p<0.01) in males, while 5% of the variation in the lesion size can be attributed to HDL cholesterol (R^2^ = 0.05, p<0.01) in females.

### QTL Analysis for the Plaque Size in the Aortic Root

**Figure 2:**
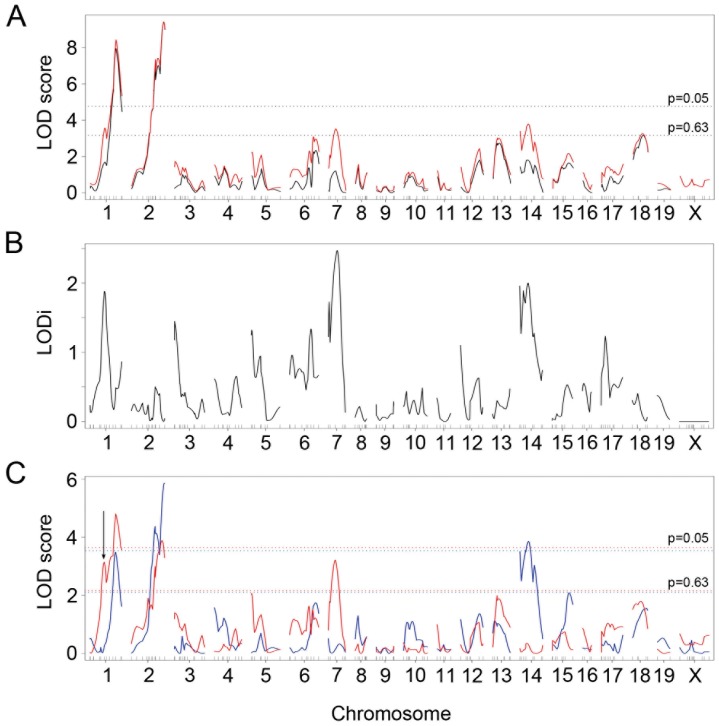
Genome-wide single QTL scans for atherosclerotic plaque size at the root. (A) LOD curves for the root lesion with sex as an additive covariate (black line) and as an interactive covariate (red line). X-axis represents positions on chromosome (cM) and y-axis represents the LOD score. The horizontal dashed lines represent the thresholds for significant QTL (p = 0.05) and suggestive QTL (p = 0.63) in the sex-interactive model. (B) Differences in LOD curves of the sex-additive model and the sex-interactive model (LODi). (C) LOD curves for root lesion in males (blue line) and females (red line). The horizontal dashed lines represent the thresholds for significant QTL (p = 0.05) and suggestive QTL (p = 0.63) in males (blue) and females (red). The arrow indicates the second QTL on Chr 1 in females.

Genome-wide scans for QTL that influence atherosclerotic plaque sizes in the aortic root and plasma lipid levels were performed using 170 informative SNP genotypes of the 309 F2 mice (172 males and 137 females). We first performed single-locus scans for root lesions using sex as an additive or an interactive covariate (Figure 2A). Significant LOD (Logarithm of the odds ratio) scores were 3.6 and 4.7 in the sex-additive (black line) and sex-interactive (red line) analyses, respectively. The detected QTL, LOD scores, 95% confidence intervals (CIs) and the variance of phenotype explained are summarized in Table 2. In the sex-interactive model, two highly significant QTL were detected on Chr 1 (LOD = 8.4, 158 Mb) and on Chr 2 (LOD = 9.4, 162 Mb). In addition, three suggestive QTL on Chr 7 (LOD = 3.5, 78 Mb), Chr 14 (LOD = 3.8, 56 Mb) and Chr 18 (LOD = 3.3, 53 Mb) were obtained (Figure 2A and Table 2). The LOD score difference between the sex-additive model and the sex-interactive model (LODi) indicates, however, that Chr 7 (LODi = 2.5) and Chr 14 (LODi = 2.0) loci interact with sex (Figure 2B). The LODi analysis also revealed an additional sex-specific locus on Chr 1 (LODi = 1.9, 75 Mb, Figure 2B). To examine a potential presence of additional QTL on Chr 1 and Chr 2, we calculated LOD score differences between the one-QTL and two-QTL models (ΔLOD) for each chromosome in the sex-interactive model. The LOD score differences showed not significant but non-negligible evidence for a second QTL at 75 Mb on Chr 1 (ΔLOD = 1.6). There was no observable evidence of a second QTL on Chr 2 (ΔLOD = 0.7).

**Table 2:** QTL for root lesion identified by genome-wide single scan.

	Chr	Peak (cM)	CI (cM)	Peak (Mb)	CI (Mb)	LOD	Significance	High Allele	Mode of Inheritance	Variance (%)
Male and Female	1	29	2–60	59	6–138	3.6	Suggestive	DBA	dominant	
	**1**	**68**	**64**–**74**	**158**	**153**–**168**	**8.4**	**Significant**	**DBA**	**recessive**	**8.8**
	**2**	**83**	**78**–**86**	**162**	**157**–**165**	**9.4**	**Significant**	**DBA**	**additive**	**8.6**
	7	41	31–50	78	55–97	3.5	Suggestive	129	dominant	1.9
	14	28	7–45	56	17–91	3.8	Suggestive	129	additive	1.2
	18	29	11–41	53	17–68	3.3	Suggestive	DBA	recessive	3.8
Male	1	68	61–77	171	162–183	3.5	Suggestive	DBA	recessive	8.0
	**2**	**85**	**60**–**86**	**172**	**139**–**173**	**5.9**	**Significant**	**DBA**	**additive**	**11.8**
	**14**	**29**	**7**–**46**	**73**	**21**–**106**	**3.9**	**Significant**	**129**	**additive**	**6.9**
Female	1	38	30–48	73	56–90	3.4	Suggestive	DBA	dominant	
	**1**	**68**	**58**–**83**	**139**	**121**–**169**	**4.8**	**Significant**	**DBA**	**recessive**	**7.0**
	**2**	**79**	**61**–**86**	**139**	**106**–**150**	**3.9**	**Significant**	**DBA**	**recessive**	**3.8**
	7	41	31–50	78	54–91	3.2	Suggestive	129	dominant	4.4

Model of inheritance was determined according to allelic effect at the nearest marker of a QTL by performing linear regression using the additive and dominant/recessive models. % Variance shows the percentage of the total F2 phenotypic variance. Significant QTL are shown in bold letters. CI, 95% confidence interval.

**Figure 3:**
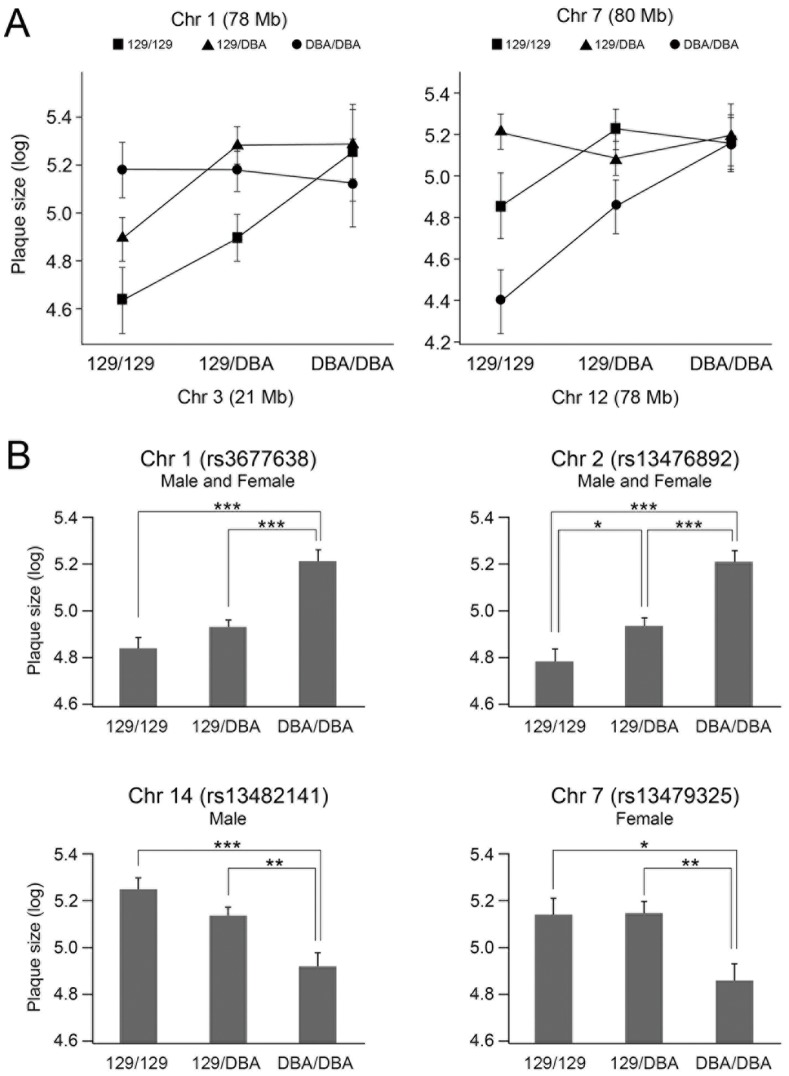
Allele effects of QTL for atherosclerosis. (A) Allelic distribution of the main effect QTL for atherosclerosis at the root in Chr 1 and Chr 2 in both sexes, Chr 14 in males, and Chr 7 in females. Atherosclerotic plaque sizes are indicated as the mean ± SEM. *p<0.05, **p<0.01, ***p<0.001. (B) The effects of interactions detected by the pair-wise genome scan on the plaque size (μm^2^) expressed in log. Interactions between Chr 1 and Chr 3 in females (left) and interactions between Chr 7 and Chr 12 in females (right).

**Figure 4:**
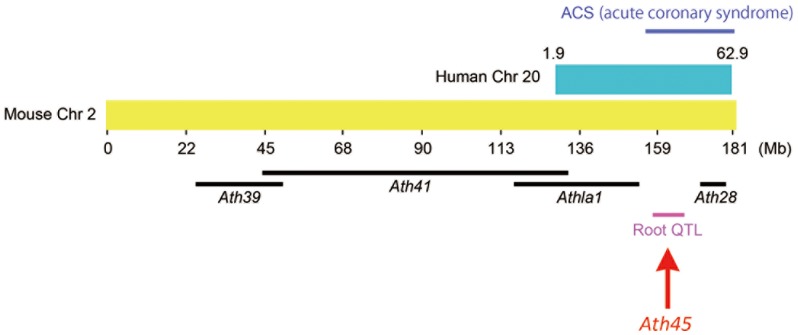
Comparative genomic map of mouse Chromosome 2. Mouse Chr 2 and homologous human Chr 20 regions are derived from the Virtual Comparative Map (VCMap) tool. The black bars represent confidence intervals of previously identified QTL for atherosclerosis; the magenta bar represents *Ath45*; the blue bar represents the human locus linked with acute coronary syndrome (ACS).

Together, our analysis revealed that two major QTL significantly affect the plaque size at the aortic root, one QTL on Chr 1 at 158 Mb and the other on Chr 2 at 162 Mb. Average size in the F2 mice homozygous for the DBA allele of the QTL on Chr 1 at 158 Mb was 2.5 times larger than those in the mice homozygous for the 129 allele of this locus, showing 129 dominance over DBA allele (Figure 3A). We name this QTL *Ath44*. Although it overlaps with previously identified *Ath1* [17], the identity is uncertain as we describe below. Similarly, average plaque size at the aortic root of F2 mice homozygous for the DBA allele of the QTL on Chr 2 at 162 Mb was 2.5 times larger than the plaque size of mice homozygous for the 129 allele of this locus. At this locus DBA and 129 show additive effects. This QTL also appears to be distinct from any of QTL previously mapped on mouse Chr 2: *Athla1* [18], *Ath28* [6], *Ath39* [19] and *Ath41* [8]. We name this novel QTL locus *Ath45*. As shown in Figure 4, *Ath45* corresponds to human chromosome 20, where a locus 20q11 - 13 has been linked with the incidence of acute coronary syndromes [20]. However, comparison between the mouse and human QTL did not reduce the interval of *Ath45*, since human 20q11 - 13 locus spans almost 35 Mb (estimated interval: 28 - 63 Mb) and include the homologous regions for *Ath41* and *Ath28*.

We next performed QTL analysis on data from each gender to confirm the sex-specific QTL. Significant LOD scores were 3.5 and 3.6 in male-only and female-only analyses, respectively. The male-only scan detected two significant QTL on Chr 2 at 172 Mb (LOD = 5.9) and on Chr 14 at 73 Mb (LOD = 3.9), and one suggestive QTL on Chr 1 (LOD = 3.5, 171 Mb) (Table 2 and Figure 2C, blue line). In the female-only scan, two significant loci, Chr 1 at 139 Mb (LOD = 4.8) and Chr 2 at 139 Mb (LOD = 3.9), and one suggestive locus on Chr 7 (LOD = 3.2, 78 Mb) were identified (Table 2 and Figure 2C, red line). ΔLOD on the female Chr 1 showed clear evidence for a second QTL on Chr 1 at around 73 Mb (ΔLOD = 3.0, arrow in Figure 2C), which was not present in the male Chr 1. The LOD curves indicate that multiple QTL may exist on Chr 2 and Chr 14 in males, and on Chr 2 in females; however, ΔLOD analysis for these chromosomes did not support this observation (ΔLOD = 0.8, 0.7, and 1.3, respectively). Thus, these sex-specific analyses: 1) identify a QTL on Chr 14 that is male-specific, 2) identify female-specific QTL on Chr 7 and Chr 1 and 3) are consistent with the results of sex-combined scan.

To better characterize the underlying genetic effect of the QTL reported above and to allow for possible epistatic interactions, we next performed genome-wide multiple QTL mapping allowing for a pair-wise scan. These results and the calculated proportion of the phenotype explained by regression analysis are shown in Table 3. In the sex-combined analysis, the best-fit model accounts for 27.9% of variation for the root lesion sizes. Chr 1 and 2 loci account for 8.2% and 8.8% of the total variance, respectively, and sex accounts for 1.9% of the total variance. The multiple-QTL model showed no evidence for significant interactions between the major effect QTL on Chr 1 and 2. In the male-only analysis, Chr 1, 2 and 14 loci account for 30.7% of the total variance and we detect no evidence of epistatic interactions. This contrasts with the results of our female-only analysis. In females, the multiple-QTL model revealed loci that were not identified by the single-locus genome scans, including QTL on Chr 3 at 21 Mb, Chr 12 at 77 Mb and Chr 17 at 67 Mb. The multiple-QTL model also revealed epistatic interactions in females between the second QTL on Chr 1 at 73 Mb and Chr 3 at 21 Mb, and between Chr 7 at 84 Mb and Chr 12 at 77 Mb. Investigation of the allelic effects of these loci identify that mice with the 129 allele at the Chr 1 locus have reduced plaque size when the animal has at least one 129 allele at the Chr 3 locus (Figure 3B). Similar results were found on Chr 7, where mice with a DBA allele at the Chr 7 locus tends to suppress the plaque size when the animal has 129 allele at Chr 12 (Figure 3B). The major effect loci (Chr 1 and 2) and sex specific and epistatic loci (Chr 1, 3, 12 and 17) jointly account for approximately 63.5% of the variance in females. The higher rate of phenotype variance explained was presumably due to more QTL mapped in females.

**Table 3:** Multiple regression analyses for root lesion in the F2 mice.

	Chromosome : cM (Mb)	Df	% Variance	F value	P value
Male and Female	Chr 1: 66 cM (156 Mb)	2	8.2	16.5	1.65E-07
	Chr 2: 85 cM (164 Mb)	2	8.8	17.6	5.98E-08
	Chr 18: 38 cM (65 Mb)	2	3.7	7.7	5.40E-04
	Sex	1	1.9	7.7	5.82E-03
	Total		27.9		
Male	Chr 1: 60 cM (160 Mb)	2	7.9	9.4	1.34E-04
	Chr 2: 73 cM (164 Mb)	2	12.1	14.4	1.73E-06
	Chr 14: 21 cM (57 Mb)	2	8.3	9.8	9.30E-05
	Total	6	30.7		
Female	Chr 1: 38 cM (73 Mb)	6	13.6	7.1	1.99E-06
	Chr 1: 74 cM (156 Mb)	2	5.9	9.2	1.96E-04
	Chr 2: 77 cM (137 Mb)	2	5.5	8.6	3.40E-04
	Chr 3: 10 cM (21 Mb)	6	14.6	7.6	7.05E-07
	Chr 7: 46 cM (84 Mb)	6	14.2	7.4	1.05E-06
	Chr 12: 39 cM (77 Mb)	6	11.6	6.0	1.67E-05
	Chr 17: 24 cM (67 Mb)	2	4.0	6.2	2.69E-03
	Chr 1: 38 cM (73 Mb)×Chr 3: 10 cM (21 Mb)	4	8.7	6.8	6.31E-05
	Chr 7: 46 cM (84 Mb)×Chr 12: 39 cM (77 Mb)	4	6.8	5.3	5.78E-04
	Total		63.5		

df, degree of freedom; % Variance shows the percentage of the total F2 phenotypic variance.

### QTL Analysis for the Plasma Lipids

Plasma levels of lipids and lipoproteins are well-established risk factors for atherosclerosis. In the current cross the QTLs for atherosclerotic plaque size are independent of those for the plasma lipid levels except for *Ath44*, which overlaps with a QTL for plasma HDL-cholesterol on Chr 1 at 126 Mb (CI: 102 - 174 Mb, LOD = 6.0) (Figure S3 and Table S2). This locus may affect lesion development through a lipid-related pathway as the 129 allele of the HDL QTL is associated with higher plasma HDL and the 129 allele of *Ath44* is protective for atherosclerosis. Two significant QTLs affecting total plasma cholesterol levels were identified by single scan analysis with sex as an interactive covariate on Chr 9 at 37 Mb (LOD = 8.6) and on Chr 11 at 83 Mb (LOD = 6.4) (Figure S3 and Table S2). Alleles of 129 at both loci conferred increased levels of total cholesterol. In contrast, at the QTL on Chr 6 at 88 Mb (LOD = 9.6), which affects plasma triglyceride levels in sex-combined and male-only analyses, DBA allele was associated with increased triglyceride levels. In addition, a locus on Chr 9 at 67 Mb was identified as a QTL for triglyceride levels in females (LOD = 5.5). None of these significant loci for plasma total cholesterol/triglyceride levels overlapped with the QTLs for the atherosclerosis.

### Candidate Intervals of the *Ath44* on Distal Chr 1

The *Ath44* localized within confidence range between 152.9 - 168.5 Mb (peak at 158.3 Mb) on Chr 1 significantly affects the lesion size at the aortic root and this region harbors more than 100 genes. To refine the candidate interval, we performed haplotype analyses in the region, followed by investigation of gene functions and gene expression levels, and further comparison with overlapping QTL.

**Figure 5:**
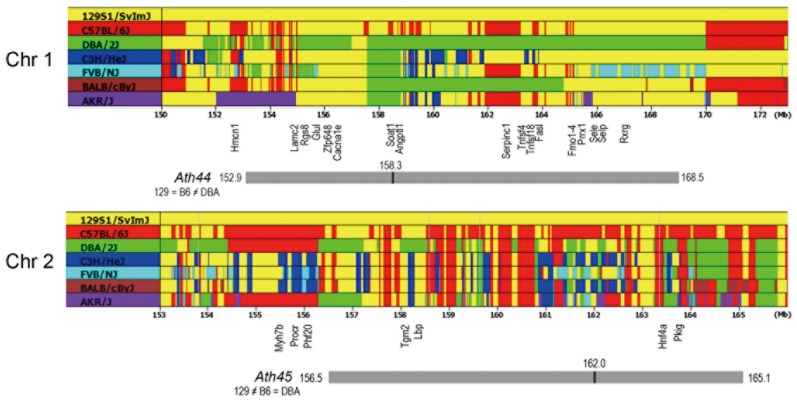
Haplotype analysis. Chromosome maps of Chr 1 and Chr 2 corresponding to *Ath44* and *Ath45* from the Parlegen Mouse SNP Browser. Sequences of 129 strain are colored by yellow and identical sequences are shown in the same color. Positions of several candidate genes are shown.

Survey of nucleotide sequence differences in the *Ath44* regions of Chr 1 between DBA/2J and 129S5, which is the closest relative to 129S6 substrain (see methods section) [21], indicates that DNA sequence of DBA differs from that of 129 extensively. Since no QTL affecting the root plaque size was present in the same region of the B6-apoE x 129-apoE cross, the simplest inference is that the candidate gene for atherosclerosis is likely to be within the blocks where 129 differs from that in DBA but shares the same haplotype with B6. Major blocks of haplotype sharing between 129S6 and C57BL/6 are 152 - 153 Mb, 155 - 157 Mb, and 160 - 168 Mb, together containing approximately 84 genes with DBA-specific SNPs that could influence at the levels of transcription/translation (Figure 5). For 50 genes with nonsynonymous SNPs, we studied each amino acid substitution for type of changes, position in the protein, homology with other vertebrate proteins, potential side chain modifications, and protein domain context. For example, flavin containing monooxygenase 3, coded by *Fmo3*, contains five amino acid substitutions unique to DBA. While four are benign changes, the substitution to Asn in DBA from highly conserved and negatively charged Asp at amino acid residue at position 76 in a NAD binding domain is predicted to be damaging when analyzed with Sorting Intolerant from Tolerant (SIFT) program with a score of 0.01 (Table S3). Similarly, there are seven amino acid substitutions between DBA and 129 E-selectin coded by *Sele*, and Ser201Phe in the first Sushi (CCP/SCR) domain was also predicted to be possibly damaging (SIFT score 0.04).

We next looked for the genes relevant to vascular development or to atherosclerosis through knockout phenotypes and human GWAS association data. The 152.9 - 168.5 Mb interval is equivalent to human Chr1q22-5. The 152 - 153 Mb region contains *Hmcn1* (hemicentin 1). Hemicentin 1 is implicated in age-related macular degeneration in humans and markers within and near *HMCN1* have been reported to be strongly associated with VLDL levels (-log_10_P = 13.9) and aortic atherosclerosis (8.9) [22]. The 155 - 157 Mb interval is syntenic with Chr1q25, where an intron SNP marker in *RGS8* is associated with cardiovascular disease (8.9) and SNPs near and within the *CACNA1E* gene are associated with HDL cholesterol levels (9.5), echocardiography (5.2) and blood pressures (4.9). The 160 - 168 Mb interval contains genes including inflammatory genes (*Tnfsf4, Tnfsf18*, *Fasl*) at 163Mb, antioxidant genes (*Fmo1 - 3*) at 165 Mb, and selectins (*Sele* and S*elp)* at 166 Mb. Differences in these inflammation related genes could influence early atherogenesis. Notable GWAS associations are *TNFSF18* with *HDL* (9.9), *DNM3* with platelet count (13.7), *SELP* with P-selectin level (60.0), and *ATP1b1* with electrocardiography (15.0).

By far the majority of GWAS data indicate that the common genetic variations affecting atherosclerosis-related vascular diseases alter gene expression levels rather than protein function. We therefore performed microarray analysis of aortic tissues from wild type mice to identify differentially expressed genes among the three parental inbred strains. Among the genes located in the 152.9 - 168.5 Mb interval, the expression of *Fmo1, Fmo2*, *Fmo3* and *Sele* was lower in DBA aorta than in B6 or 129 aortas, while the expression of *Npl* and *ATP1b1* was higher (Table 4 and Figure S4). We also estimated gene expression levels in peritoneal macrophages from parental strains by exploratory microarray. In macrophages from DBA mice, the expression of *Npl, Tnfsf4,* and *ATP1b1* was higher than in macrophages from 129 mice (Table 4 and Figure S5).

**Table 4:** Haplotype analysis of *Ath44* on Chr 1.

			Aortic expression			Macrophage expression					
Gene	Mb	Haplotype	DBA/129	B6/129	Level	DBA/129	B6/129	Level	AAsubstitution	human mutation, GWAS (−log_10_P)	KO/mutant mice phenotype
Hmcn1	152.4	129≠B6, Bc, C, F≠D≠A	1.6	0.9	19	0.9	0.7	14	K957E, Q1183H, I5571V, G5607R	VLDL (13.9), atrial fibrillation(5.7), aorta (8.9), CRP (7.2)	
Lamc1	155.1	129, B6, A, C, Bc≠D, F	1.0	**1.3** ^b^	1481	1.2	0.6	657	None	CAD (5.4)	embryonic death
Npl	155.3	129, B6, A, C, Bc ≠ D, F	**4.8** ^c^	1.4	40	5.9	0.6	289	None	HDL (5.9)	
Rgs8	155.5	129, B6, A, C, Bc ≠ D, F	0.9	0.8	20	1.0	1.1	26	None	CVD (8.8)	normal development
Glul	155.7	129, B6, A, C, Bc ≠ D, F	0.5	0.9	3883	1.1	0.6	2060	None	cholesterol (9.6)	embryonic lethal
Cacna1e	156.2	129, B6, A, Bc, C, F ≠ D	0.7	0.8	24	0.9	1.0	25	G710E	HDL (9.5), echocardiography(5.2), BP (4.9)	impaired glucose tolerance
Soat1	158.3	129, B6 ≠ D, A, Bc, C, F	1.1	1.1	360	1.0	1.4	3087	I147V, H205Y		protected from diet induced hypercholesterolemia
Tor3a	158.6	129, B6 ≠ D, A, Bc, C, F	**1.5** ^a^	1.4	169	1.5	1.3	751	R94H, **R119C**		
Fam20b	158.6	129, B6 ≠ D, A, Bc, C, F	**1.2** ^a^	0.9	692	1.4	0.8	753	None	BMI (4.2)	
Angptl1	158.8	129, B6 ≠ D, A, Bc, C, F	1.8	1.1	38	0.9	0.7	13	P279T, N318K	insulin (4.0)	normal
Serpinc1	162.9	129, C ≠ B6, A, F ≠ D, Bc	1.2	6.7	14	0.9	1.4	14	L376I	thrombophilia	prenatal death, heterozygotes have increased thrombosis
Tnfsf4	163.3	129, B6, A, C, F ≠ D, Bc	1.0	0.8	9	2.9	0.7	19	None	lupus (31.5)	decreased T cell proliferation
Tnfsf18	163.4	129, B6, A, C, F ≠ D, Bc	1.1	1.5	11	1.1	0.8	11	T157N	HDL (9.9)	
Fasl	163.4	129, B6, A, C, F ≠ D, Bc	1.2	1.2	9	1.0	1.1	8	T184A, E218G	BP (4.1)	enlarged lymph and spleen
Pigc	163.9	129, A, C ≠ B6, F ≠ D, Bc	**0.7** ^b^	0.8	56	1.1	1.1	57	None	associated with sCD14 levels	
Dnm3	163.9	129, A, C ≠ B6, F ≠ D, Bc	**1.4** ^a^	1.1	49	0.8	0.8	25	None	waist-hip ratio (17.0), plateletcount (13.7), inter-adventitialcommon carotid artery diameter	normal
Fmo1	164.7	129, B6, A, C, F ≠ D, Bc	**0.4** ^b^	0.8	1850	0.9	1.0	37	None	sudden cardiac death (5.7)	
Fmo2	164.8	129, B6, A, C, F ≠ D, Bc	**0.5** ^c^	0.9	4898	1.1	0.7	21	None	None	
Fmo3	164.9	129, B6, A, Bc, C, F ≠ D	**0.1** ^c^	**0.6** ^b^	3870	1.4	1.1	17	**D76N**, N118D, A201S, M318V, L526I	trimethylaminuria	
Prrx1	165.1	129, C, Bc ≠ B6, A, F ≠ D	1.3	0.8	1327	1.5	0.5	113	None	mutations cause agnathia-otocephaly complex	neonatal death, abnormal aortic arch and vascular development
Sele	165.9	129, B6, A, Bc, C, F ≠ D	**0.8** ^a^	1.0	45	0.9	0.8	32	R12H, V87A, I89V, **S201F**, P206L, P352S, S391N		increased infection
Selp	166.0	129, B6, A, Bc, C, F ≠ D	0.4	1.3	224	0.3	0.1	712	None	P-selectin (60.0)	abnormal leukocyte rolling
Slc19a2	166.2	129, B6, A, Bc, C, F ≠ D	0.8	1.0	121	1.3	0.9	142	None	megaloblastic anemia, TG (9.9)	abnormal blood cells, develop diabetes mellitus on thiamine-free diet
Atp1b1	166.4	129, B6, A, Bc, C, F ≠ D	**2.0** ^a^	1.2	687	1.9	0.7	58	None	electrocardiography(15.0), BMI (5.7)	

Genes with DBA-unique sequences (DBA ≠ 129 = B6) within and near the interval of 152.9 - 168.5 Mb are shown. For each gene, expression levels in the aorta and macrophages, amino acid (AA) differences (129, B6-position-DBA), associations with atherosclerosis indicated by GWAS data, and phenotypes of knockout mice are shown. Ratios between two strains that show significant difference in the expression are bolded. AA substitutions that are predicted to be deleterious by SIFT program are bolded (see table S3). ^a^ p<0.05, ^b^ p<0.01, ^c^ p<0.001. A, AKR; Bc, Balb/c; B6, C57BL/6; C, C3H; D, DBA; F, FVB.

Since SNPs within the given haplotype blocks of interest could affect the expression of the genes at far distance away from them, we next screened aortic expression QTL (eQTL) data from the Hybrid Mouse Diversity Panel (HMDP) [23] for associations (Table S4). SNPs were selected according to the criteria: 1) located within and near the QTL interval, 2) associated with genes at P<1.00E-06, and 3) having nucleotide sharing pattern of DBA ≠ 129 = B6. Majority of SNPs associated with the expression of genes peaked nearby to them (cis) confirming strain-specific differences in their regulations, although not all resulted in overt changes in aortic or macrophage expression in our experiments. Some SNPs affect the genes at >10 Mb away on the same chromosome, including *Sox13*, *Ifi202*, *Ephx1* and *Agfg1*. Among these SNPs, rs31529590 at 158.1 Mb is strongly associated with the expression of *Ifi202b* at 175.9 Mb. On the other hand, there are multiple other cis-acting SNPs for *Ifi202b*, where nucleotides are shared in 129 and DBA but different in B6. This raises a possibility that two different regions regulate this gene, one unique in DBA and the other shared in 129 and DBA. Indeed, expression levels of *Ifi202b* are highest in DBA and lowest in B6. Additionally, the analyses revealed that SNPs within *Ath44* interval appear to be affecting 16 genes on different chromosomes. Many of the trans-eQTLs peak at rs32041876 located within the *Dnm3* gene where multiple non-coding short transcripts including mirRNAs have been detected. However, none of these associate genes showed DBA-specific expression in the aorta.

Collectively, we prioritized candidate genes underling *Ath44* on Chr 1 by analyses of haplotype blocks, surveys of gene functions and expressions, and an analysis of an eQTL panel; 24 genes (Table 4) and additional 2 genes with eQTL (Table S4) were selected to be most likely responsible for plaques in the aortic root area.

### Candidate Intervals of *Ath45* on Chr 2

The DBA allele of *Ath45* at 162.0 Mb (CI: 156.5 - 165.1 Mb) is associated with larger aortic root plaques significantly in the DBA-apoE x 129-apoE cross. Our previous B6-apoE x 129-apoE cross showed a suggestive QTL at the same location (peak: 154 Mb, CI: 112 - 164 Mb, LOD: 3.8) where B6 allele confers atherosclerosis susceptibility over 129 allele [15], suggesting that the locus is likely shared in DBA and B6. Consequently, regions where 129 haplotypes differ from those which are shared by DBA and B6 were further considered. The haplotype-sharing pattern of 129 ≠ DBA = B6 occurs in 42 genes (Figure 5). The features of these genes analyzed using the same strategies described above are summarized in Table 5.

**Table 5:** Haplotype analysis of *Ath45* on Chr 2.

			Aortic expression			Macrophage expression					
Gene	Mb	Haplotype	DBA/129	B6/129	Level	DBA/129	B6/129	Level	AAsubstitution	human mutation,GWAS (−log_10_P)	KO/mutant mice phenotype
Phf20	156.1	129 = Bc, C, F ≠ B6, D, A	**0.7** ^a^	0.8	295	0.9	1.2	298	N248S, S344		craniofacial defect, low plasma glucose
Tgm2	158.0	129 ≠ B6, A, Bc, C, F ≠ D	1.0	0.9	3304	0.8	0.5	2105	None		disorganized myocardium
Lbp	158.1	129, A, Bc, C, F ≠ B6, D	1.1	0.8	389	1.7	1.8	80	**G25C**, S102R, **Y284H**	heart rate (4.9)	increased gram-negative bacterial infection
Plcg1	160.5	129 ≠ B6, A, Bc, C, F, D	1.0	1.1	612	1.0	0.9	272	None		embryonic lethal, abnormal vasculogenesis
Ptprt	161.5	129 ≠ B6, A, Bc, C, F, D	0.9	0.9	15	1.0	1.3	13	None	kidney disease (9.0)	increased tumorigenesis
Sgk2	162.8	129 ≠ B6, A, Bc, C, F, D	1.2	**1.7** ^a^	11	1.0	0.9	18	None		abnormal hair growth
Ift52	162.8	129 ≠ B6, A, Bc, C, F, D	1.1	1.1	213	1.1	1.2	362	**N22I**		preimplantation death
Mybl2	162.9	129 ≠ B6, A, Bc, C, F, D	**0.7** ^a^	0.8	54	1.0	0.8	78	None		embryonic lethal
Hnf4a	163.3	129 ≠ B6, A, Bc, C, F, D	1.3	2.5	15	1.1	1.2	18	None	Type2 diabetes (9.5), HDL (15.0)	preweaning death
Serinc3	163.4	129 ≠ B6, A, Bc, C, F, D	**1.2** ^a^	1.2	5155	1.3	1.4	3508	V162F, S403N		
Pkig	163.5	129 ≠ B6, A, Bc, C, F, D	**1.5** ^a^	1.2	51	1.5	1.8	28	G52A, S70N		embryonic death, abnormal heart
Ada	163.5	129 ≠ B6, A, Bc, C, F, D	**0.4** ^b^	**0.5** ^b^	161	3.3	0.9	80	None	immunodeficiency, tunica media (4.8)	hepatocellular impairment, perinatal lethality
Stk4	163.9	129 ≠ B6, A, Bc, C, F, D	0.8	0.8	302	1.1	0.9	699	None	T-cell immunodeficiency, recurrent infections and cardiac malformations	abnormal leukocyte migration
Sdc4	164.2	129 ≠ B6, A, Bc, C, F, D	0.9	0.9	1712	1.1	0.6	501	None	LDL (4.2), E-selectin (4.8)	decreased angiogenesis
Elmo2	165.1	129 ≠ B6, A, Bc, C, F, D	1.1	1.1	499	1.0	1.1	934	N547S		
Slc2a10	165.3	129 ≠ B6, A, Bc, C, F, D	1.2	1.2	137	0.9	1.1	35	None	arterial tortuosity syndrome	G128E mutation abnormal artery morphology

Genes with 129-unique sequences (129 ≠ B6 = DBA) within and near the interval of 156.5 – 165.1 Mb are shown. For each gene, expression levels in the aorta and macrophages, amino acid (AA) differences (DBA, B6-position-129), associations with atherosclerosis indicated by GWAS data, and phenotypes of knockout mice are shown. Ratios between two strains that show significant difference in the expression are bolded. AA substitutions that are predicted to be deleterious by SIFT program are bolded (see table S3). ^a^ p<0.05,^ b^ p<0.01. A, AKR; Bc, Balb/c; B6, C57BL/6; C, C3H; D, DBA; F, FVB.

Although the syntenic region in humans has not been associated with atherosclerosis, the region (157.8 - 158.3 Mb) includes the functional candidate *Lbp*, which encodes lipopolysaccharide binding protein. By interacting with CD14, Lbp plays key roles in innate immune signaling pathways, including toll-like receptor signaling pathway. Lbp protein of 129 contains Gly25Cys, and Tyr284His substitutions compared to the proteins in B6 and DBA. Gly-25 is a conserved residue at the signal peptide cleavage site and the change to Cys-25 could alter processing of the matured protein. Additionally, Tyr-284 is in the conserved lipid binding domain and its change to His-284 could alter its function. Each change is predicted to be deleterious with SIFT score of 0.01and 0.03, respectively (Table S3). *Lbp* also showed reduced expression in 129 macrophages compared to DBA and B6 (Table 5 and Figure S5), although further confirmations are required.

In the 160 - 164 Mb interval, an Asn22Ile substitution in the *Ift52* gene coding for an intraflagellar transport protein essential for the formation and maintenance of flagella and cilia is potentially damaging with a SIFT score of 0.03 (Table S3). Asn-22 is a conserved residue within an ABC-type uncharacterized transport system domain, which is found in various eukaryotic and prokaryotic intra-flagellar transport proteins. *Pkig*, coding for protein kinase inhibitor gamma, showed lower aortic expression in 129 than in DBA. Pkig protein of 129 also has two amino acid substitutions compared to Pkig proteins in B6 and DBA, Gly52Ala and Ser70Asn. Both Gly-52 and Ser-70 are highly conserved during evolution. Gly52Ala substitution is possibly damaging (SIFT score 0.08), while Ser70Asn might be benign (Table S3). Aortic expression of *Ada,* which encodes adenosine deaminase, is expressed more than two times higher in 129 than in DBA and B6. ADA plays an important role in purine metabolism and adenosine homeostasis, thereby modulates signaling of extracellular adenosine and T-cell activation. While ADA deficiency causes autosomal recessive SCID (severely combined immunodeficiency) due to toxic effects of substrate accumulation, a lower activity of the enzyme appears to be associated with reduced incidence of CAD in humans [24] and may be beneficial in controlling intimal formation as SMC-derived adenosine can inhibit cell proliferation in culture [25]. Higher *Ada* gene expression in the aorta of 129 mice (Table 5 and Figure S6), however, cannot simply account for their reduced susceptibility to atherosclerosis. Additionally, although no nonsynonymous SNPs or gene expression difference were observed, human GWAS has indicated strong associations of a missense mutation and an intron variant in *HNF4a* with plasma HDL levels (-log_10_P = 15.0) and type 2 diabetes (-log_10_P = 9.5), respectively. Additionally, decreased angiogenesis was observed in mice lacking syndecan 4 coded by *Sdc4* [26].

Finally, eQTL data of HMDP panels provided 6 additional genes in the *Ath45* interval, expression of which is associated with SNPs at P<1.00E-06, having nucleotide sharing pattern of 129 ≠ B6 = DBA (Table S5). Of these 6 genes, *Plac9* on chromosome 14 and *Exosc1* on chromosome 19 were affected in trans. Microarray data showed *Dynlrb1* and *Plac9* have lower expression in 129 compared to DBA and B6, although their association with atherosclerosis has not been reported. Together, we reduced the candidate interval of *Ath45* on Chr 2 according to haplotype blocks, gene functions and gene expression levels, suggesting 16 genes and additional 2 genes with eQTL as the putative candidates for the root atherosclerosis.

## Discussion

In the present study, we identified multiple QTLs that significantly affect atherosclerotic plaque size in the root area in an F2 population derived from a cross between DBA-apoE and 129-apoE. We carried out genome analysis of two QTLs, *Ath44* and *Ath45,* that are significant in male and female combined analyses, by combining the current QTL information with information obtained from our previous B6-apoE x 129-apoE cross. *Ath44* on Chr 1 overlaps with *Ath1*, but they are likely to be distinct, while *Ath45* on the Chr 2 is a novel locus.

A large number of loci have been mapped in mice for atherosclerotic plaque development, and some of these loci overlap. However, whether these loci are identical or discrete is difficult to determine since experiments carried out in different laboratories have used different designs, such as husbandry, types of diet, use of sensitizing mutations, sex and lineage of cross, which likely affect the quantitative phenotype [27], [28]. QTL information from crosses using apoE-deficient mice in six different combinations of seven laboratory inbred backgrounds have been reported including our current work; four of them involves C57BL/6J partnered with C3H/HeJ, BALB/cJ, FVB/NJ or 129S6, and the remaining two involves DBA/2J, partnered with 129S6 or AKR/J. While the information derived from these multiple crosses should help define the regions responsible for the QTL, there are several notable discrepancies. The best example is *Ath1*on Chr 1. *Ath1* was originally identified to affect diet-induced atherosclerosis in the aortic root of the female mice of recombinant inbred strains between C57BL6/J and C3H/HeJ, and between C57BL6/J and BALB/cJ [17]. *Tnfsf4* (*Ox40l*) at 163Mb has been suggested as the gene underlying this locus [9]. *Tnfsf4* encodes a ligand of OX40, which is a member of the tumor necrosis factor (TNF) receptor superfamily. In females, the mRNA level of *Tnfsf4* was 3.7 times higher in atherosclerosis-susceptible C57BL/6J mice than in atherosclerosis-resistant C3H/HeJ mice [9], and the Tnfsf4-OX40 signaling is proposed to affect the susceptibility of atherosclerosis by modulating T cell proliferation [9]. *Ath1* was subsequently mapped at 158 Mb (CI: 135 -161 Mb) in the analysis of the F2 population from C57BL/6J-apoE x C3H/HeJ-apoE intercross [29], and at 186Mb (CI: 178 - 190 Mb) in C57BL/6J-apoE x BALB/cJ-apoE intercross [8]. In both of these crosses, mice were fed a Western-type diet containing high fat/high cholesterol, and *Ath1* was not replicated in chow-fed C57BL/6J-apoE x C3H/HeJ-apoE [30] nor in a cross of LDLR^−/−^ mice on C57BL/6J and BALB/cByJ backgrounds fed a diet low in cholesterol [19]. *Ath1*, therefore, appears to be restricted to studies where a cholic acid containing diet was used to exacerbate atherosclerosis. Thus crosses that use genetic susceptibility in the form of apoE^−/−^ and LDLR^−/−^, or cholic acid-containing high fat/high cholesterol diets may yield different loci, reflecting distinct susceptibility alleles and underlying mechanisms.

The *Ath44* overlaps with *Ath1*. However, while the B6 allele at the *Ath1* locus conferred increased atherosclerosis compared to both the C3H/HeJ and BALB/cJ alleles, the DBA allele of *Ath44* was associated with increased atherosclerosis compared to either the B6 or 129 alleles. Although the variations within or near *Tnfsf4* could be responsible for the increased atherosclerosis susceptibility of DBA over 129 in our cross, they cannot explain both *Ath1* and *Ath44* since DBA shares an identical DNA sequence in and near *Tnfsf4* with the atherosclerosis-resistant BALB/cJ mice, while 129S6 and C3H/HeJ share the same sequence with the atherosclerosis-susceptible C57BL/6J allele. In addition, *Ath44* was detected in both male-only and female-only analysis in our study, whereas the effect of *Tnfsf4* variance appears to be female-specific. Furthermore, our DBA-apoE x 129-apoE F2 mice were on regular chow. It appears likely that multiple loci within 150 - 170 Mb are affecting root atherosclerosis, and that one or more of these loci could be interacting with either a diet containing high cholesterol or cholic acid.

Several other groups have performed crosses of apoE-deficient mice on low cholesterol diets and not replicated the *Ath1* locus. For example a DBA-apoE x AKR-apoE cross did not detect a significant QTL near *Ath1* [6], [31], and only one of the two groups of B6-apoE x FVB-apoE cross analyzed detected a QTL distal to the *Ath1* locus at 178 Mb [5]. The haplotype sharing pattern of the interval 157.5 – 159 Mb, where both FVB/NJ and AKR/J share DNA sequence with DBA2 but differ from 129 and B6, is consistent with these observations. Furthermore, while C3H/HeJ and BALB/c also share haplotypes with DBA/2J not with C57BL/6J in this region, but there is no indication that their alleles cause larger plaques than the C57BL/6J allele. Thus, these crosses of apoE^−/−^ mice suggest, although not conclusively, that 157.5 – 159 Mb is an unlikely candidate region. Instead, the genetic changes responsible for increased plaque size in the root of DBA mice are more likely to be within the intervals where DBA sequence uniquely differs from others. The region containing a cluster of flavin containing monooxygenase genes, *Fmo1*, *Fmo2* and *Fmo3* is particularly interesting because these genes are highly expressed in aortic tissues, their expression is significantly low in DBA compared to 129 and B6, and *Fmo3* contains a potentially damaging amino acid substitution. A recent report identified a SNP in DBA mice that affects an enhancer site for farnesoid X receptor (Fxr/Nr1h4) binding [32]. Although this may not be the causal SNP for atherosclerosis, these results suggest a possible mechanism for altered *Fmo3* expression [32]. Whether and how reduced function of these Fmos affects increased risk for plaque size at the aortic root in DBA require further investigation, since *Rgs8*, *Glul, Cacna1e, Prrx1, Sele, Selp* and *Atp1b1* that affect vascular health are also within these regions.

As to the *Ath45* on Chr 2, the SNPs within and near most of the genes in the 157 – 164 Mb interval are unique to 129 and other strains all share SNPs with DBA, which is consistent with the absence of obvious QTLs observed in crosses of apoE-null mice on other strain combinations than those involving 129-apoE. One of the primary candidates is *Lbp*, in which two amino acid substitutions predict a reduced function of the 129 protein compared to those in DBA and B6. Athero-protective role of the 129 Lbp is supported by the observed association between serum Lbp levels and CAD in humans [33] and well-documented links among chronic infections, inflammation, and atherosclerosis, as well as roles of LBD, CD14 and TLR in atherosclerosis [34]. Although further evidence is required, *Pkig* is another primary candidate in the 160 – 164 Mb interval, which encodes c-AMP dependent protein kinase inhibitor gamma. While its role in vascular disease is not yet to be determined, *Pkig* is expressed in many tissues including heart and endothelial cells [35], [36]. Our observation of a 30% reduction in the *Pkig* expression in aortic tissues of 129 mice compared to DBA mice as well as amino acid changes in the highly conserved residues of 129 Pkig protein compared to DBA proteins warrants further examination of their functional relevance. *Hnf4a*, coding for hepatic nuclear factor 4, is also located in *Ath45*, although expression difference in parental strains was not detectable and no amino acid changes are present in the protein sequences. As a transcription factor, Hnf4a regulates expression of many genes in atherosclerosis-prone aortas of mice [37]. Two QTL for plasma triglyceride, *Tgq31* and *Tgq32* [38], and two QTL for HDL cholesterol, *Hdlq19* [39] and *Hdlq70* [38], have been mapped to the overlapping region with *Ath45* and *Hnf4a* has been proposed as an HDL cholesterol associated gene [40]. However, we did not detect any QTL for plasma TG or HDL cholesterol on Chr 2 in the present 129×DBA intercross. Thus, if *Hnf4a* is contributing to the athero-protection in the aortic root of 129-apoE mice, its role is likely to be independent of the mechanisms that control plasma lipid concentration.

While comparative genome analysis of inbred mouse stains is a powerful approach, there are limitations in our study. One is that the confidence interval estimated by the Bayesian method could be too stringent. For example, *Ath45* peak in males extends further to the end of the chromosome and conservative estimation of confidence interval with one-LOD drop suggests that *Ath45* could overlap with *Ath28* previously found in a DBA/2J and AKR/J cross [6]. If each QTL contains multiple causative genes, the haplotype sharing pattern may be more complex than the assumption we made in our analyses. Another limitation is that the Sanger genome project includes complete genome sequence of 129S1 and 129S5 but 129S6. However, 129S6 strain was derived in 1992 from a cross between 129S5 and 129/SvEv-Gpi1^c^, both established in 1987-1988 from a colony of 129S1 (129/SvEv) maintained by M.J. Evans since 1969 [21]. The genome of each inbred laboratory strain of mice is a unique mosaic of old polymorphisms between the parental chromosomes from different subspecies of *Mus musculus*. Fixation of new variations in the inbred strains during the last 100 years or so since their derivation has been minimum [41]. Thus the probability is small that a new nucleotide change arose in these chromosomal regions of our interest, has been fixed in the 129S6 population, and is functionally relevant. Nevertheless, possible presence of SNPs recently arose in the parental lines of apoE-deficient strains cannot be eliminated completely.

Our current study also revealed the presence of gender-specific QTLs. The QTL on Chr 14 is significant only in males and the 129 allele confers the susceptibility over DBA. It peaks at 73 Mb with a broad confidence interval of 21 - 106 Mb, and co-localizes with *Ath13* that was previously identified in males of B6-apoE x FVB-apoE cross [5]. Our QTL may well be identical to *Ath13* since both were detected in the male-only and the sex-combined analyses but not in the female-only scans. Female-specific epistatic interactions between a second locus on Chr 1 at 73 Mb and Chr 3 at 21 Mb, and between Chr 7 at 84 Mb and Chr 12 at 77 Mb have not been reported and require further investigations. Nevertheless, the QTL on the Chr 1 at 73 Mb may well be the same as *Ath30*, which was identified by B6-apoE x C3H-apoE cross [29] and was also female-specific. A proteoglycan desmin encoded by *Des* has been implicated in atherosclerosis in humans [42] and has also been proposed as a candidate gene underlying *Ath30* [23]. Similarly, Chr 7 locus co-localizes with QTL, *Ath3* [43] and *Aorls2* [44] previously identified in wild type female mice fed an atherogenic diet from crosses between B6 x DBA/2, A/J x B6, respectively. It also overlaps with and may well be identical to *Ath3,* which was identified as a female-specific QTL in a B6-apoE x C3H-apoE cross [29]. We note that the QTL region on Chr 7 contains imprinting domains including one equivalent to Prader-Willi/Angelman syndromes gene cluster on human Chr 15. Epigenetic interactions make comparative genome analysis difficult since the absence of QTL in a certain cross no longer provides simple information. Nevertheless, even though each locus accounts for a small portion of the phenotype variations, accumulations and interactions of such minor factors substantially contribute to the individual susceptibility to atherosclerosis. The interactions between QTLs and the differences between males and females could also provide important keys to identify the underlying genes.

In conclusion, with the use of publicly available mouse genome information and published data of crosses of apoE null mice on various genetic backgrounds, we suggest that the *Ath44* and *Ath45* are likely to be in 155 - 157 Mb or 165.3 - 168 Mb intervals on Chr 1 and in 160 - 164 Mb on Chr 2. Candidate genes of 25 or so in each locus were further reduced to suggest several genes for future investigation of how these loci influence atherosclerosis susceptibility at the aortic root of mice. These genes include, *Fmo3, Sel*e, and *Selp* for *Ath44,* and *Lbp* and *Pkig* for *Ath45*, and multiple SNPs within the equivalent regions in human genome have been shown to associate with cardiovascular phenotypes. While determining the causalities still requires a long process, and the causative genes in mice and humans may differ, searching genetic variations that influence atherosclerotic plaque development with multiple inbred strains of mice would likely lead to the mechanistic studies of known associations as well as to identification of novel genes/pathways involved in the pathogenesis of atherosclerosis. This information in turn should have important implications for prevention and treatment of atherosclerosis.

## Materials and Methods

### Animals

ApoE-deficient mice on a 129S6/SvEvTac genetic background (129-apoE) were generated in our laboratory as previously described [4], [15]. ApoE-deficient mice on a DBA2J background (DBA-apoE) were obtained from the Jackson Laboratory (#007067, D2.129P2 (B6)-apoE^tm1Unc^/J). Male 129-apoE mice were mated to female DBA-apoE mice to generate F1 hybrids, which were intercrossed to generate F2 progeny. Mice were fed with a regular mouse chow (Prolab IsoPro RMH 3000; Agway Inc) and handled in strict accordance with the recommendations in the Guide for the Care and Use of Laboratory Animals of the National Institutes of Health under protocols approved by the Institutional Animal Care and Use Committees of the University of North Carolina at Chapel Hill (Protocol Number: 11-028).

### Phenotyping

Atherosclerotic plaque sizes were measured as previously described [4], [15]. Briefly, mice at 4 months of age were anesthetized with a lethal dose of 2,2,2-tribromoethanol, perfused with 4% paraformaldehyde, and the heart and the aorta were dissected. Atherosclerotic lesions in the roots were evaluated by sectioning the proximal aorta and the heart containing the aortic root. Plaque areas were measured on the captured images using Image J 1.43 software (http://rsb.info.nih.gov/ij/). Plasma levels of total cholesterol and triglycerides were measured using commercial kits from Wako Chemicals USA (Richmond, VA) and Stanbio Laboratory (Boerne, TX), respectively. HDL cholesterol levels were determined after removing apoB containing lipoproteins with magnesium/dextran sulfate [45].

### DNA Isolation and Genotyping

Genomic DNA was isolated from the livers and purified using DNeasy Tissue Kit (Qiagen, Hilden, Germany), followed by quantification using PicoGreen dsDNA Assay Kit (Molecular Probes, Eugene OR, USA). Single nucleotide polymorphism (SNP) genotyping was performed using the Illumina BeadArray technology with Mouse Low Density Linkage SNP panels, which is an optimized set of 377 SNPs covering the 19 autosomes and the X chromosome and includes approximately four SNPs per each 27 Mb interval across the entire mouse genome.

### QTL Analysis

We performed QTL analyses using R/qtl program (v.1.26-14, http://www.rqtl.org) [46], [47]. The main effect QTL were identified by genome-wide single-QTL scans using the ‘*scanone*’ function with standard interval mapping. Genotype probabilities between markers were computed with 1 cM intervals and with a genotyping error rate of 0.001. The significance thresholds were determined by 10,000 permutations. QTL were considered significant if LOD scores exceeded 95% (p<0.05) of the permutation distribution; they were considered suggestive if the scores exceed 37% (p<0.63) distribution. 95% confidence intervals (CIs) for QTL positions were calculated by Bayesian credible interval function of the software. To determine sex-specific QTL, main scans were performed with sex as an additive covariate or as an interactive covariate. If the LOD score difference between the two models (LODi) was greater than 2, the sex-QTL interaction was considered to be significant [48]. We also performed QTL analysis in males and females separately. The presence of two QTL on the same chromosome was assessed by comparing two models: a model with one QTL and a model with both QTL. If the LOD score difference (ΔLOD) was greater than 2, two QTL were considered to be present on the same chromosome [48]. For each QTL, model of inheritance was determined according to allelic effect at the nearest marker of a QTL by performing linear regression using the additive and dominant/recessive models.

Multiple-QTL models were detected by the ‘*stepwiseqtl*’ function of R/qtl, which perform a forward/backward stepwise search algorithm to select the best-fitting model. Penalties for the model comparison corresponding to α = 0.1 were calculated using the result of 1,000 permutations with the ‘*scantwo*’ function. The proportion of variance explained and LOD score under the model were calculated using the ‘*fitqtl*’ function. A multiple imputation was used for the analyses. All the genetic positions (cM) were converted to physical positions (Mb, NCBI Build 37) using the Mouse Map Converter (http://cgd.jax.org/mousemapconverter/).

### Microarray Analysis

Aortas were dissected from 3- to 5-month-old male mice. Peritoneal macrophages were isolated from 3-month-old mice of both sexes 4 days after the intraperitoneal injection of 1 ml thioglycollate. Tissues/macrophages from 4-5 mice were pooled and total RNA was extracted using an RNeasy Mini Kit (QIAGEN), according to manufacturer’s protocol. RNA quality was assessed by an Agilent Bioanalyzer. Total RNA (250 ng) was used to synthesize fragmented and labeled sense-strand cDNA and hybridize onto Affymetrix arrays. The Affymetrix HT WT User Manual was followed to prepare the samples. Briefly, the WT Expression HT Kit for Robotics (Ambion) was used to generate sense-strand cDNA from total RNA. Following synthesis of sense-strand cDNA, the cDNA was fragmented and labeled with the Affymetrix GeneChip HT Terminal Labeling Kit. The Beckman Coulter Biomek FXP Laboratory Automation Workstation with the Target Express set up was used to prepare the samples with these two kits. Fragmented and labeled cDNA was used to prepare a hybridization cocktail with the Affymetrix GeneTitan Hybridization Wash and Stain Kit for WT Arrays. Hybridization, washing, staining and scanning of the Affymetrix array plate was carried out using the Affymetrix GeneTitan MC Instrument. GeneChip Command Console Software (AGCC) was used for GeneTitan Instrument control. Affymetrix Expression Console Software was used for basic data analysis and quality control. All the samples were processed using a single Mouse Gene 2.1 ST 24-Array Plate (Affymetrix), except for comparison between 129 and DBA macrophages, which was carried out at a different time using Mouse Gene 2.0 ST Array (Affymetrix). The array data was deposited to Gene Expression Omnibus (GEO) Database (accession number GSE53006).

### Comparative Genomics and Haplotype Analysis

SNPs and nucleotide sequence comparisons of mouse strains were obtained from publicly available resources (http://www.sanger.ac.uk/resources/mouse/genomes/, http://useast.ensembl.org/Mus_musculus/, and http://www.informatics.jax.org/). Sanger genome project includes whole genome sequence of 129S1 and 129S5 but 129S6. However, 129S6 strain was derived in 1999 from a cross between 129S5 and 129/SvEv-Gpi1^c^, two substrains of a 129S1 strain maintained by M.J. Evans (129/SvEv) since 1969 [21]. No nucleotide differences were detected in the relevant regions. Consequently, we used 129S5 sequence to represent 129S6 sequence in our comparisons. The haplotype patterns of genomes in the QTL interval were analyzed using UNC Mouse Phylogeny Viewer (http://msub.csbio.unc.edu/) and Perlegen Mouse SNP Browser (http://mouse.cs.ucla.edu/perlegen/). The likelihood that an amino acid change is detrimental to a protein was examined by Sorting Intolerant From Tolerant (SIFT) program (http://sift.jcvi.org/) [49]. Information of human GWAS data was obtained from NHGRI GWAS catalog (http://www.genome.gov/gwastudies/). Gene expression data in the aorta of mice in the Hybrid Mouse Diversity Panel (HMDP) [21] was obtained from GEO Database (accession number GSE38120). SNPs were selected from the eQTL data of the HMDP according to the criteria: 1) located within and near the QTL interval, 2) associated with genes at P<1.00E-06, and 3) having nucleotide sharing pattern of DBA ≠ 129 = B6 (*Ath44*) or 129 ≠ B6 = DBA (*Ath45*). Clusters of SNPs linked were represented by one with smallest P value, followed by the shortest distance to the start of the gene when it is on the same chromosome. All the genomic locations are in Mb.

### Statistical Analysis

Data were analyzed using JMP software version 8.0 (SAS Institute, Cary, NC). Comparisons of multiple groups in trait values and gene expression levels in the aorta were done by one-way analysis of variance (ANOVA) followed by Tukey-Kramer’s HSD test. The distributions of phenotypes in F2 mice were assessed for normality by Shapiro–Wilk test and Kolmogorov test. For gene expressions in macrophages, the genes in *Ath44* or *Ath45* did not differ by sex. The fold differences were therefore the average of male and female samples. At least two independent pools of samples for each genotype were analyzed and their average was used to estimate relative ratios and level of gene expression for the present study.

## Supporting Information

Figure S1 **Distributions of the plasma lipids.** Histograms of log-transformed plasma lipid concentration (total cholesterol, HDL cholesterol, and triglyceride) in 340 F2 mice derived from 129-apoE and DBA-apoE mice.
(TIF)

Figure S2 **Correlations between root plaque sizes and plasma lipids.** Correlations of root plaque sizes with T-Chol, HDL-C, and TG in F2 males and females.
(TIF)

Figure S3 **Genome-wide single QTL scans for plasma lipids.** LOD curves for plasma lipids by single QTL scans in both sexes with sex as an interactive covariate (left), in males (middle) and in females (right). The horizontal dashed lines represent the thresholds for significant QTL (p = 0.05) and suggestive QTL (p = 0.63).
(TIF)

Figure S4 **Expression levels of candidate genes for** ***Ath44*** **in the aorta of DBA, 129 and B6.** Aortic gene expression levels in the male aorta of each strain were detected by microarray analysis (n = 3). Values are shown as the mean ± SD.
(TIF)

Figure S5 **Expression levels of candidate genes in the macrophages of DBA, 129 and B6.** Gene expression levels in the macrophages were compared between DBA and 129 (left), and 129 and B6 (right) by microarray analyses. In each comparison, values of males (closed rectangle) and females (open circle) were normalized to 129-male.
(TIF)

Figure S6 **Expression levels of candidate genes for** ***Ath45*** **in the aorta of DBA, 129 and B6.** Aortic gene expression levels in the male aorta of each strain were detected by microarray analysis (n = 3). Values are shown as the mean ± SD.
(TIF)

Table S1 **Normality tests for phenotype distributions.** Distributions of each phenotype in the F2 population (n>300, male and female combined) were assessed for normality with a Shapiro-wilk test using the original (non-transformed) values, and with a Kolmogorov -Smirnov test using the log-transformed values.
(DOCX)

Table S2 QTL for plasma lipids identified by genome-wide single scan. CI, 95% confidence interval; Significant QTL are shown in bold letters.
(DOCX)

Table S3 **Estimated effects of amino acid substitutions.** Effects of amino acid (AA) substitutions were predicted by SIFT (Sorting Intolerant From Tolerant) program. SIFT scores show the probability that an amino acid change is damaging, ranged from 0 to 1. AA substitutions with SIFT score ≤0.05 are predicted to be deleterious; substitutions with SIFT score >0.05 to be tolerated.
(DOCX)

Table S4 **SNPs within the** ***Ath44*** **interval associated with gene expressions in the aorta.** SNPs within the 152.9 - 168.5 Mb interval of Chr 1, having nucleotide sharing pattern of 129 = B6 ≠ DBA and associated with genes at *P*<1.00E-06 were selected from the eQTL data of the Hybrid Mouse Diversity Panel (HMDP) [21]. Those in linkage disequilibrium are represented by a single SNP with lowest *P*-value and nearest to the associated gene represents. Distance is the position of SNP site relative to the start position of the gene. For each SNP, expression levels of the associated genes in the aorta and macrophages are shown as ratios of two strains. Genes that show DBA-specific expression are bolded. ^a^p<0.05, ^b^p<0.01, ^c^p<0.001.
(DOC)

Table S5 **SNPs within the** ***Ath45*** **interval associated with gene expressions in the aorta.** SNPs within the 156.5 - 165.1 Mb interval of Chr 2, having nucleotide sharing pattern of 129 ≠ B6 = DBA, and associate with genes at *P*<1.00E-06 were selected from the eQTL data of the Hybrid Mouse Diversity Panel (HMDP) [21]. A SNP with lowest *P*-value and nearest to the associated gene represents those in linkage disequilibrium. Distance is the position of SNP site relative to the start position of the gene. For each SNP, expression levels of the associated genes in the aorta and macrophages are shown as ratios of two strains. ^a^p<0.05, ^b^p<0.01, ^c^p<0.001. Genes that show 129-specific expression are bolded.
(DOC)
