# Supplementary figures and images for: Quantitative Trait Loci Affecting Atherosclerosis at the Aortic Root Identified in an Intercross between DBA2J and 129S6 Apolipoprotein E-Null Mice
Source: PLoS One. 2014 Feb 20;9(2):e88274. doi: 10.1371/journal.pone.0088274 (PMC3930552; doi:10.1371/journal.pone.0088274)

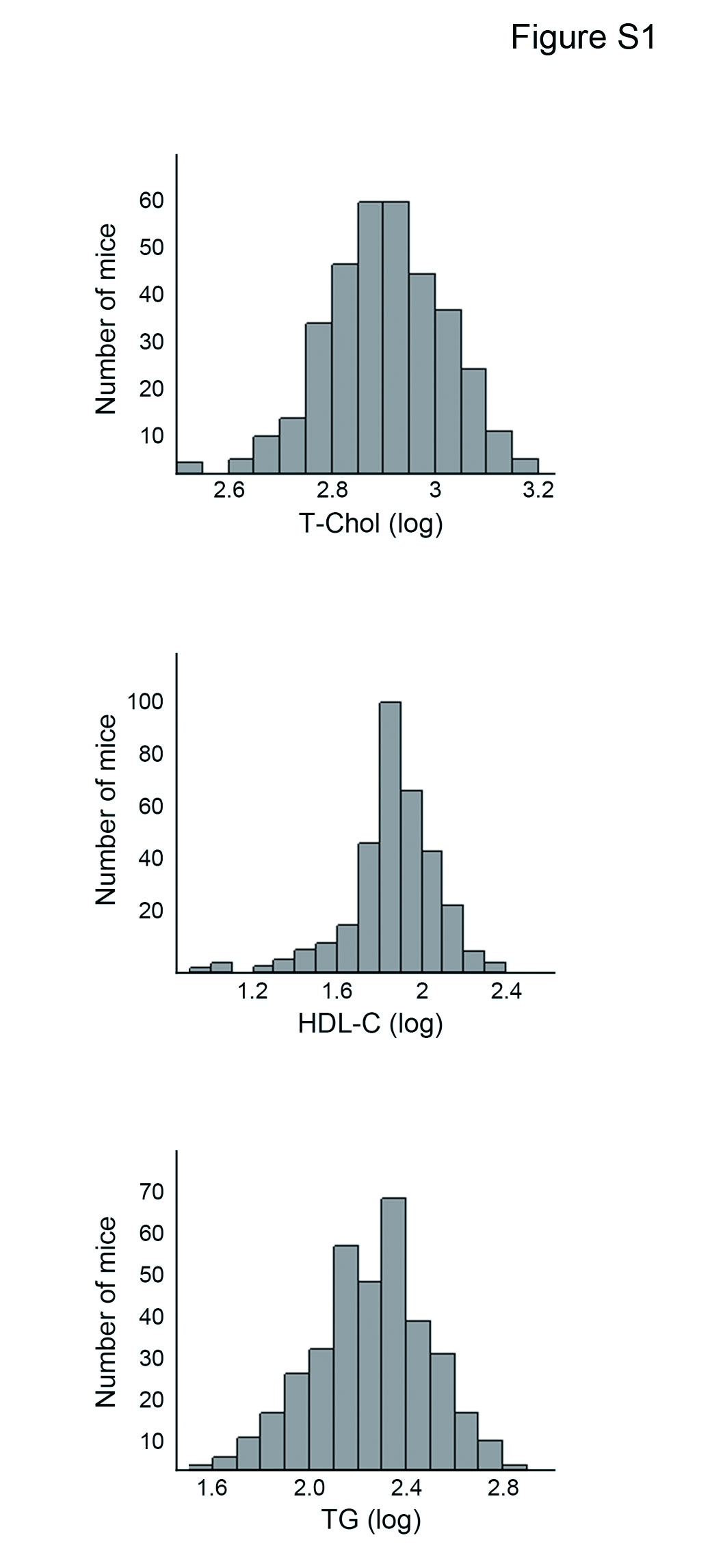

Supplement: Figure S1 — Distributions of the plasma lipids. Histograms of log-transformed plasma lipid concentration (total cholesterol, HDL cholesterol, and triglyceride) in 340 F2 mice derived from 129-apoE and DBA-apoE mice. (TIF) [file pone.0088274.s001.tif]

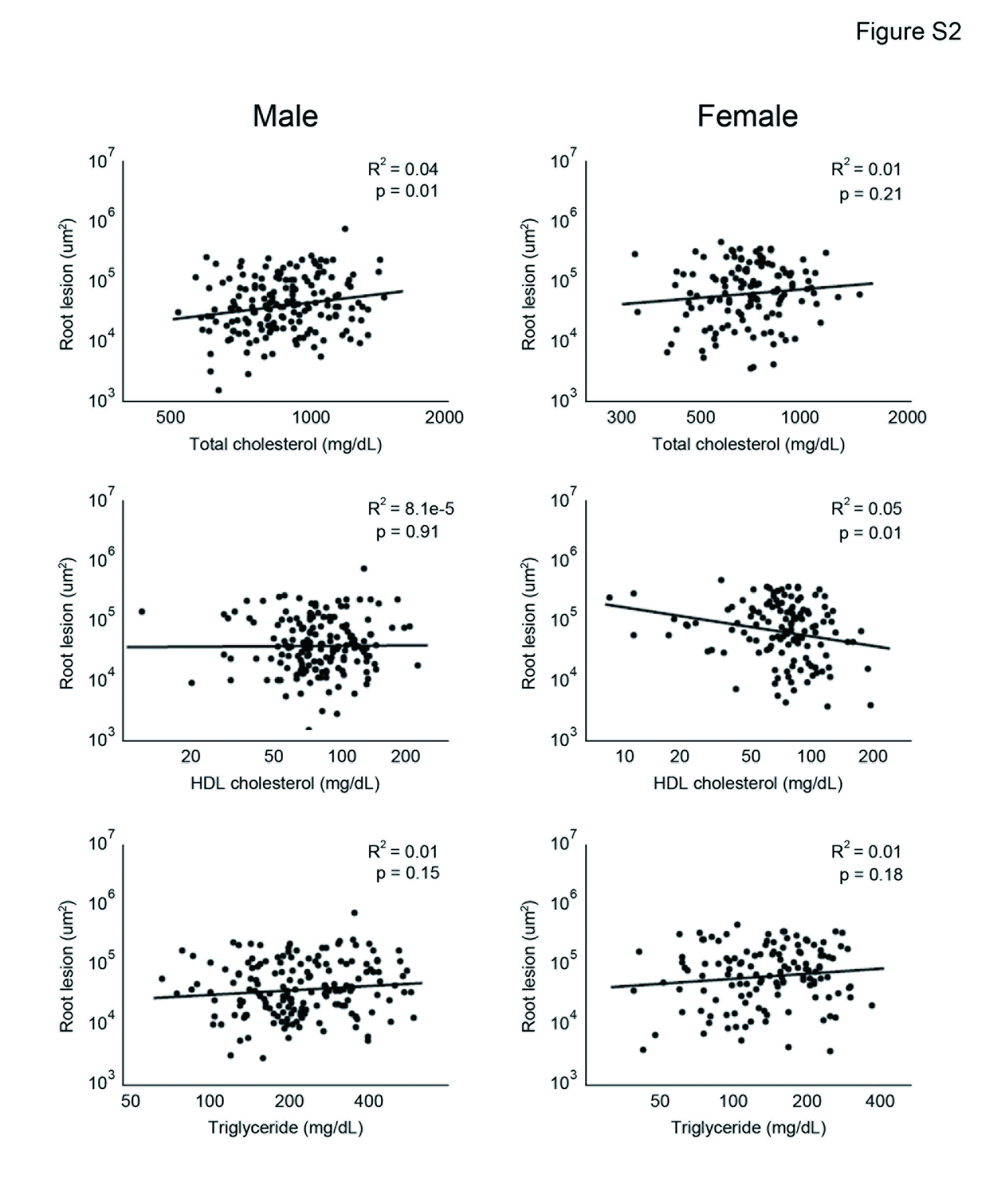

Supplement: Figure S2 — Correlations between root plaque sizes and plasma lipids. Correlations of root plaque sizes with T-Chol, HDL-C, and TG in F2 males and females. (TIF) [file pone.0088274.s002.tif]

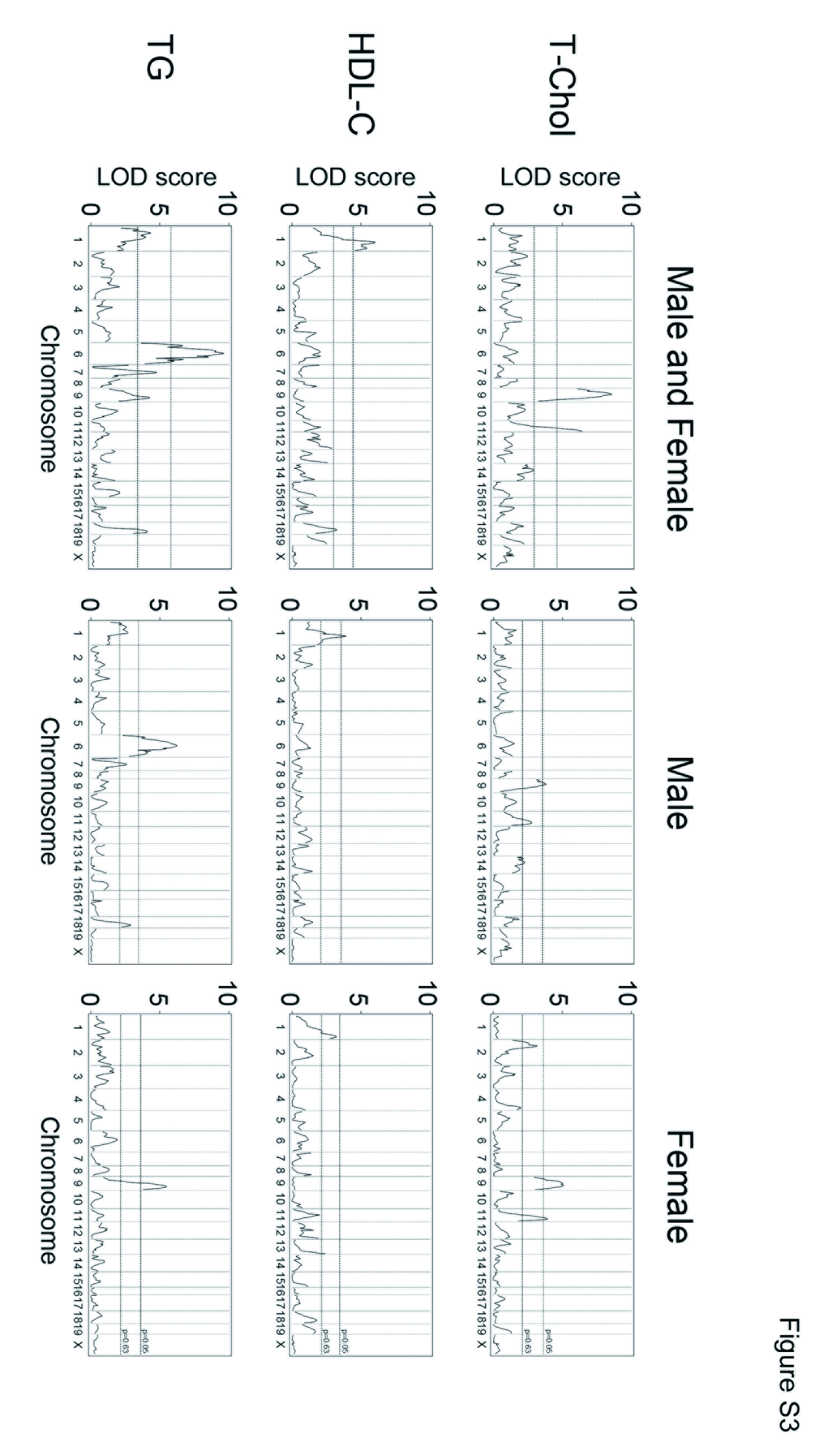

Supplement: Figure S3 — Genome-wide single QTL scans for plasma lipids. LOD curves for plasma lipids by single QTL scans in both sexes with sex as an interactive covariate (left), in males (middle) and in females (right). The horizontal dashed lines represent the thresholds for significant QTL (p = 0.05) and suggestive QTL (p = 0.63). (TIF) [file pone.0088274.s003.tif]

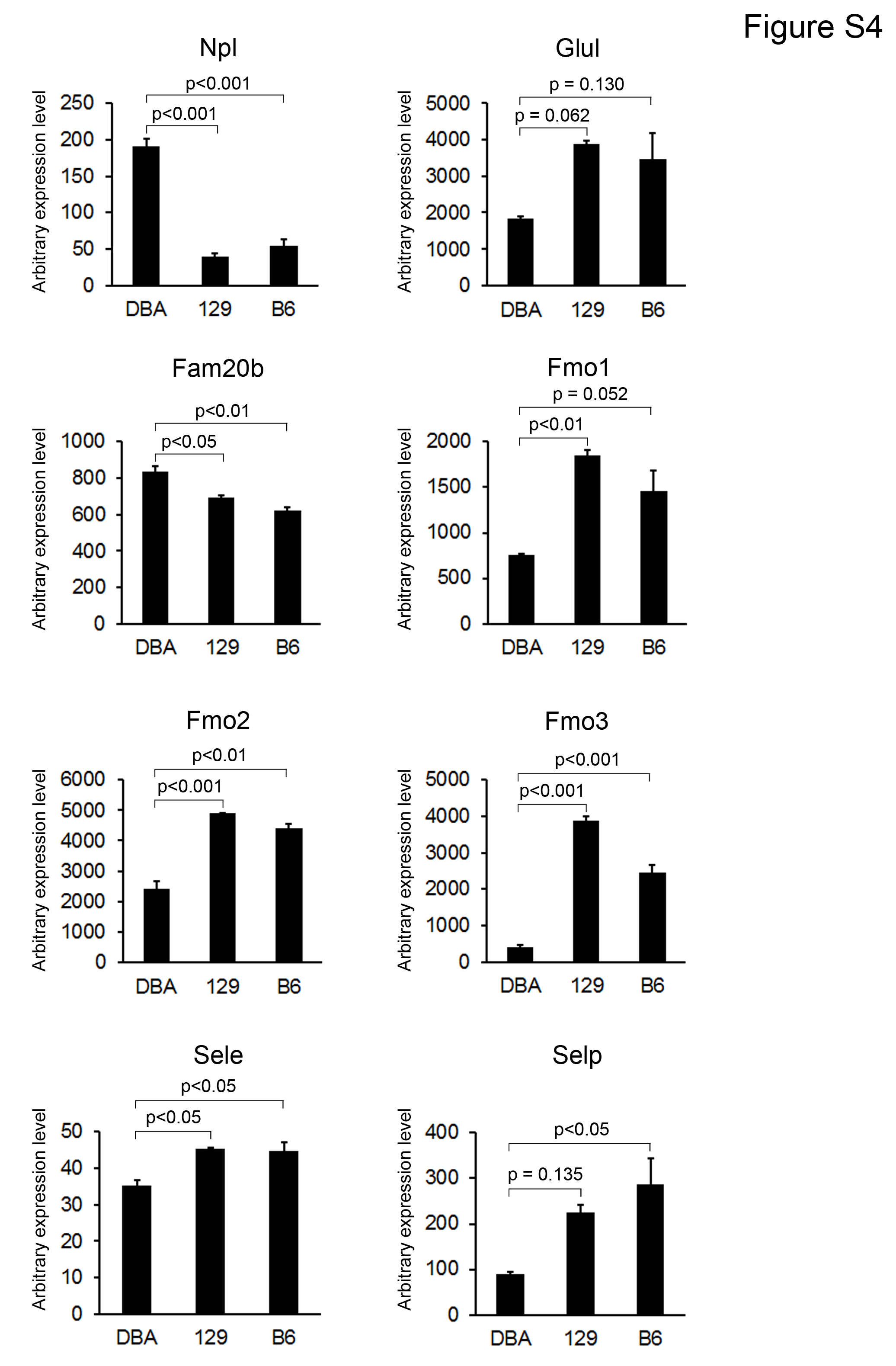

Supplement: Figure S4 — Expression levels of candidate genes for Ath44 in the aorta of DBA, 129 and B6. Aortic gene expression levels in the male aorta of each strain were detected by microarray analysis (n = 3). Values are shown as the mean ± SD. (TIF) [file pone.0088274.s004.tif]

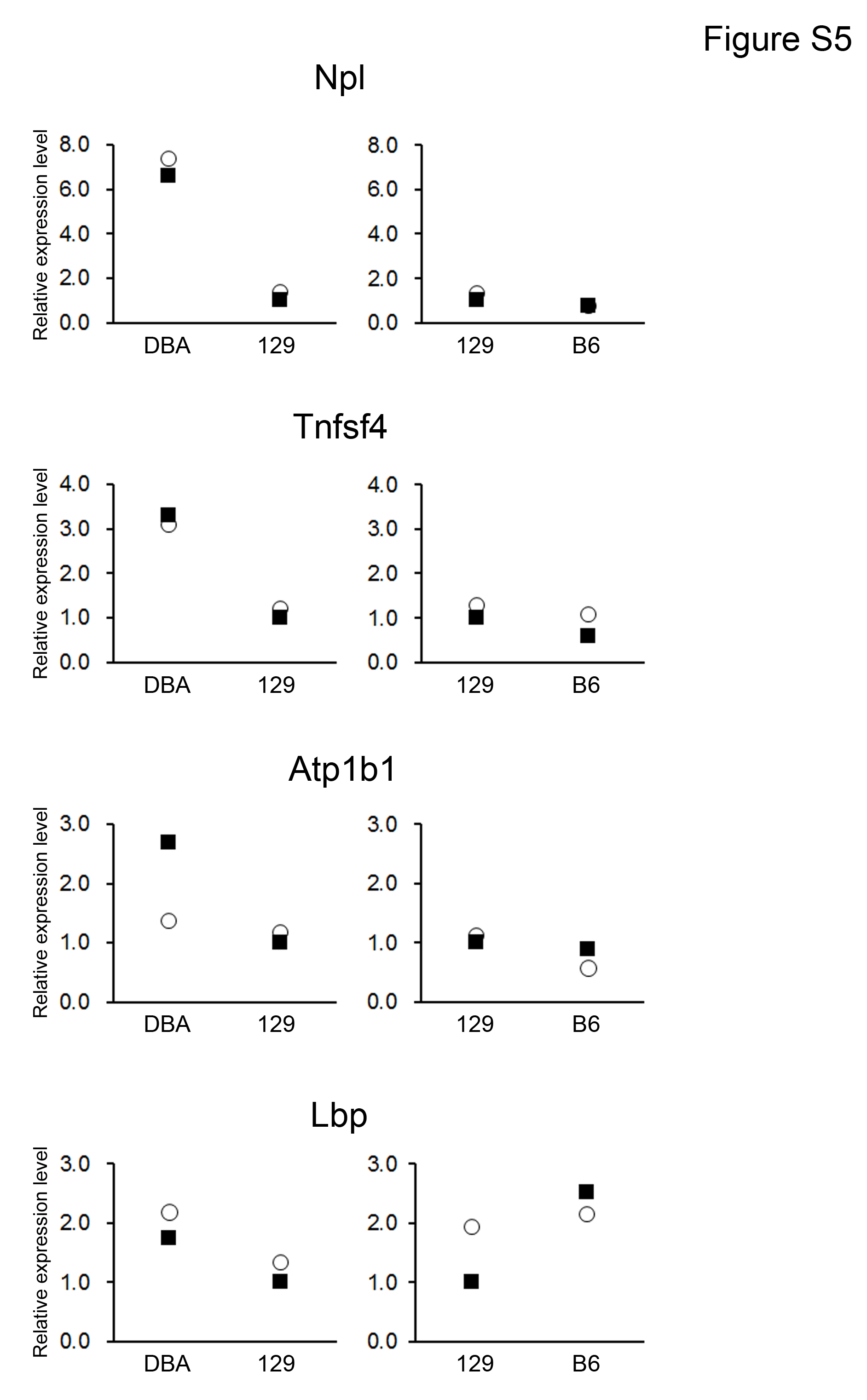

Supplement: Figure S5 — Expression levels of candidate genes in the macrophages of DBA, 129 and B6. Gene expression levels in the macrophages were compared between DBA and 129 (left), and 129 and B6 (right) by microarray analyses. In each comparison, values of males (closed rectangle) and females (open circle) were normalized to 129-male. (TIF) [file pone.0088274.s005.tif]

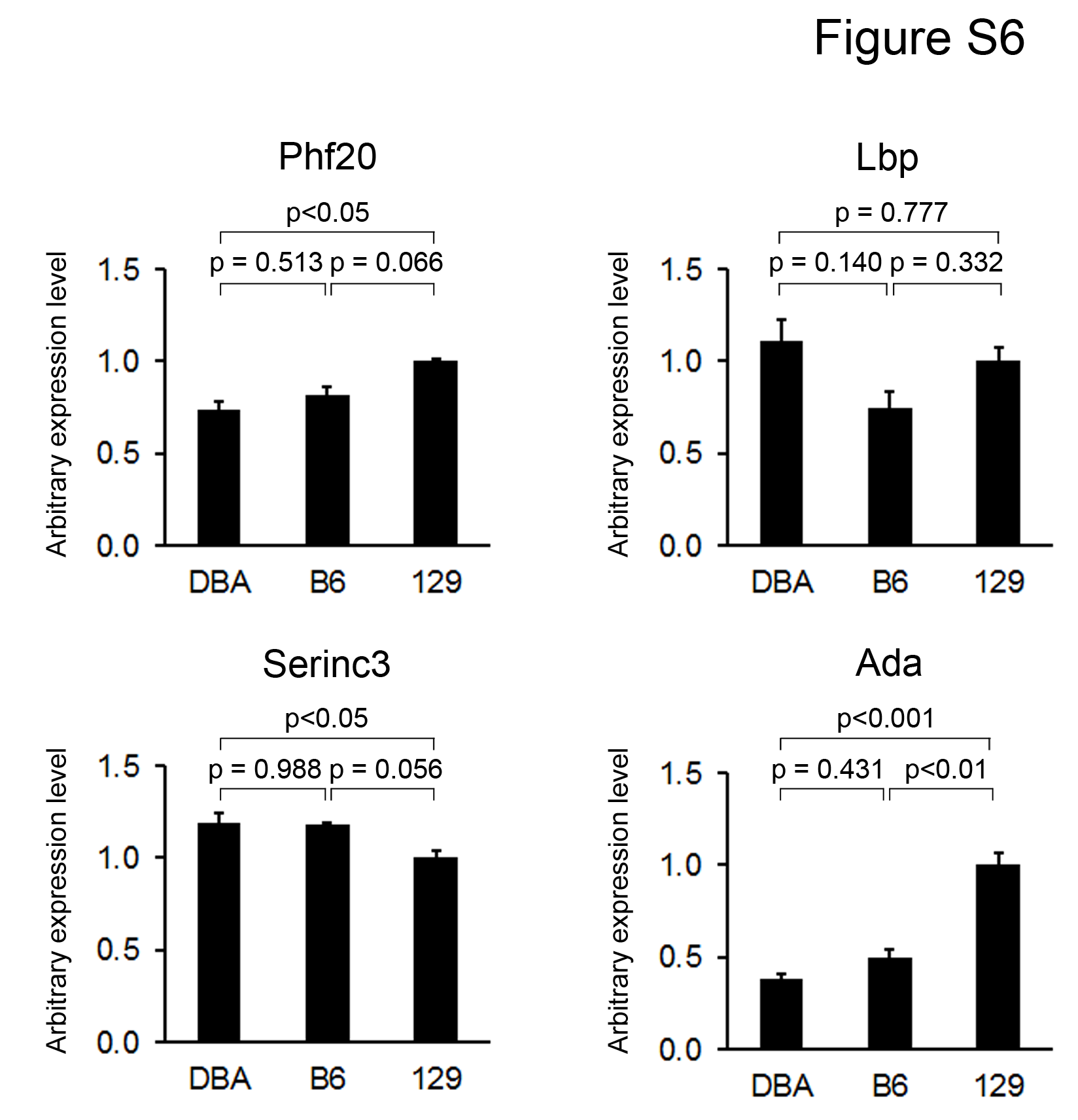

Supplement: Figure S6 — Expression levels of candidate genes for Ath45 in the aorta of DBA, 129 and B6. Aortic gene expression levels in the male aorta of each strain were detected by microarray analysis (n = 3). Values are shown as the mean ± SD. (TIF) [file pone.0088274.s006.tif]
