# Supplementary material for: Quantitative Trait Loci Affecting Atherosclerosis at the Aortic Root Identified in an Intercross between DBA2J and 129S6 Apolipoprotein E-Null Mice
Source: PLoS One. 2014 Feb 20;9(2):e88274. doi: 10.1371/journal.pone.0088274 (PMC3930552; doi:10.1371/journal.pone.0088274)
Supplement: Table S1 — Normality tests for phenotype distributions. Distributions of each phenotype in the F2 population (n>300, male and female combined) were assessed for normality with a Shapiro-wilk test using the original (non-transformed) values, and with a Kolmogorov -Smirnov test using the log-transformed values. (DOCX) [file pone.0088274.s007.docx]

**Table S1. Normality tests for phenotype distributions**

|  | Normal distribution (Shapiro-wilk test) | | Log distribution (Kolmogorov test) | |
| --- | --- | --- | --- | --- |
| Phenotype | W | P | D | P |
| Root lesion | 0.790 | < 0.0001 | 0.042 | 0.150 |
| T-Chol | 0.978 | < 0.0001 | 0.028 | 0.150 |
| HDL | 0.916 | < 0.0001 | 0.045 | 0.089 |
| TG | 0.918 | < 0.0001 | 0.044 | 0.084 |
