# Supplementary material for: Quantitative Trait Loci Affecting Atherosclerosis at the Aortic Root Identified in an Intercross between DBA2J and 129S6 Apolipoprotein E-Null Mice
Source: PLoS One. 2014 Feb 20;9(2):e88274. doi: 10.1371/journal.pone.0088274 (PMC3930552; doi:10.1371/journal.pone.0088274)
Supplement: Table S2 — QTL for plasma lipids identified by genome-wide single scan. CI, 95% confidence interval; Significant QTL are shown in bold letters. (DOCX) [file pone.0088274.s008.docx]

**Table S2. QTL for plasma lipids identified by genome-wide single scan.**

|  |  | Chr | Peak (cM) | CI (cM) | Peak (Mb) | CI (Mb) | LOD | Significance | High Allele | Variance (%) |
| --- | --- | --- | --- | --- | --- | --- | --- | --- | --- | --- |
| T-Chol | Male and Female | **9** | **21** | **15-31** | **37** | **29-57** | **8.6** | **Significant** | **129** | **7.2** |
|  |  | **11** | **50** | **43-50** | **83** | **71-83** | **6.4** | **Significant** | **129** | **6.4** |
|  | Male | **9** | **16** | **2-26** | **39** | **13-56** | **3.8** | **Significant** | **129** | **6.1** |
|  |  | 11 | 49 | 33-55 | 83 | 58-93 | 2.8 | Suggestive | 129 | 5.4 |
|  |  | 14 | 25 | 5-55 | 62 | 20-120 | 2.3 | Suggestive | 129 | 3.5 |
|  | Female | 2 | 25 | 5-31 | 30 | 5-43 | 3.2 | Suggestive | DBA | 7.0 |
|  |  | **9** | **35** | **17-45** | **58** | **28-75** | **5.1** | **Significant** | **129** | **15.9** |
|  |  | **11** | **55** | **45-59** | **90** | **70-92** | **3.9** | **Significant** | **129** | **12.7** |
| HDL-C | Male and Female | **1** | **54** | **48-80** | **126** | **102-174** | **6.0** | **Significant** | **129** | **6.9** |
|  |  | 18 | 29 | 21-39 | 53 | 39-66 | 3.2 | Suggestive | 129 | 2.2 |
|  | Male | **1** | **48** | **36-64** | **126** | **71-167** | **3.9** | **Significant** | **129** | **9.1** |
|  | Female | 1 | 82 | 57-91 | 168 | 118-179 | 3.2 | Suggestive | 129 | 6.7 |
|  |  | 13 | 56 | 24-56 | 91 | 39-91 | 2.4 | Suggestive | 129 | 5.4 |
| TG | Male and Female | 1 | 20 | 6-44 | 41 | 19-88 | 4.3 | Suggestive | 129 | 2.6 |
|  |  | **6** | **39** | **27-47** | **88** | **54-100** | **9.6** | **Significant** | **DBA** | **8.4** |
|  |  | 7 | 49 | 45-53 | 95 | 87-104 | 4.8 | Suggestive | - | 0.4 |
|  |  | 9 | 35 | 23-43 | 64 | 41-77 | 4.3 | Suggestive | 129 | 3.0 |
|  |  | 18 | 35 | 29-41 | 62 | 53-69 | 4.1 | Suggestive | - | 0.7 |
|  | Male | 1 | 34 | 2-56 | 66 | 5-154 | 2.7 | Suggestive | 129 | 5.3 |
|  |  | **6** | **36** | **22-46** | **92** | **54-110** | **6.2** | **Significant** | **DBA** | **9.8** |
|  |  | 7 | 45 | 25-53 | 89 | 38-114 | 2.6 | Suggestive | - | 3.1 |
|  |  | 18 | 30 | 22-36 | 65 | 57-69 | 2.9 | Suggestive | DBA | 4.2 |
|  | Female | **9** | **39** | **31-49** | **67** | **49-81** | **5.5** | **Significant** | **129** | **13.5** |

CI, 95% confidence interval; Significant QTL are shown in bold letters.
