# Supplementary material for: Quantitative Trait Loci Affecting Atherosclerosis at the Aortic Root Identified in an Intercross between DBA2J and 129S6 Apolipoprotein E-Null Mice
Source: PLoS One. 2014 Feb 20;9(2):e88274. doi: 10.1371/journal.pone.0088274 (PMC3930552; doi:10.1371/journal.pone.0088274)
Supplement: Table S3 — Estimated effects of amino acid substitutions. Effects of amino acid (AA) substitutions were predicted by SIFT (Sorting Intolerant From Tolerant) program. SIFT scores show the probability that an amino acid change is damaging, ranged from 0 to 1. AA substitutions with SIFT score ≤0.05 are predicted to be deleterious; substitutions with SIFT score >0.05 to be tolerated. (DOCX) [file pone.0088274.s009.docx]

**Table S3. Estimated effects of amino acid substitutions.**

| QTL | Chr | Gene | AA substitution | SIFT score | Effect |
| --- | --- | --- | --- | --- | --- |
| Ath44 | 1 | Hmcn1 | K957E | 1 | tolerated |
|  |  |  | Q1183H | 0.16 | tolerated |
|  |  |  | I5571V | 1 | tolerated |
|  |  |  | G5607R | 1 | tolerated |
|  | 1 | Cacna1e | G710E | 0.35 | tolerated |
|  | 1 | Soat1 | I147V | 0.56 | tolerated |
|  |  |  | H205Y | 0.93 | tolerated |
|  | 1 | Tor3a | R94H | 0.37 | tolerated |
|  |  |  | **R119C** | **0.02** | **deleterious** |
|  | 1 | Serpinc1 | L376I | 0.36 | tolerated |
|  | 1 | Tnfsf18 | T157N | 1 | tolerated |
|  | 1 | Fasl | T184A | 0.09 | tolerated |
|  |  |  | E218G | 0.23 | tolerated |
|  | 1 | Fmo3 | **D76N** | **0.01** | **deleterious** |
|  |  |  | N118D | 1 | tolerated |
|  |  |  | A201S | 0.1 | tolerated |
|  |  |  | M318V | 1 | tolerated |
|  |  |  | L526I | 0.21 | tolerated |
|  | 1 | Sele | R12H | 0.57 | tolerated |
|  |  |  | V87A | 0.14 | tolerated |
|  |  |  | I89V | 0.49 | tolerated |
|  |  |  | **S201F** | **0.04** | **deleterious** |
|  |  |  | P206L | 0.28 | tolerated |
|  |  |  | P352S | 0.87 | tolerated |
|  |  |  | S391N | 0.55 | tolerated |
| Ath45 | 2 | Phf20 | N248S | 1 | tolerated |
|  |  |  | S344 | 0.81 | tolerated |
|  | 2 | Lbp | **G25C** | **0.01** | **deleterious** |
|  |  |  | S102R | 0.63 | tolerated |
|  |  |  | **Y284H** | **0.03** | **deleterious** |
|  | 2 | Ift52 | **N22I** | **0.03** | **deleterious** |
|  | 2 | Serinc3 | V162F | 0.09 | tolerated |
|  |  |  | S403N | 0.22 | tolerated |
|  | 2 | Pkig | G52A | 0.08 | tolerated |
|  |  |  | S70N | 0.71 | tolerated |
|  | 2 | Elmo2 | N547S | 0.38 | tolerated |

Effects of amino acid (AA) substitutions were predicted by SIFT (Sorting Intolerant From Tolerant) program. SIFT scores show the probability that an amino acid change is damaging, ranged from 0 to 1. AA substitutions with SIFT score ≤0.05 are predicted to be deleterious; substitutions with SIFT score >0.05 to be tolerated.
