# Supplementary material for: Quantitative Trait Loci Affecting Atherosclerosis at the Aortic Root Identified in an Intercross between DBA2J and 129S6 Apolipoprotein E-Null Mice
Source: PLoS One. 2014 Feb 20;9(2):e88274. doi: 10.1371/journal.pone.0088274 (PMC3930552; doi:10.1371/journal.pone.0088274)
Supplement: Table S4 — SNPs within the Ath44 interval associated with gene expressions in the aorta. SNPs within the 152.9 - 168.5 Mb interval of Chr 1, having nucleotide sharing pattern of 129 = B6 ≠ DBA and associated with genes at P<1.00E-06 were selected from the eQTL data of the Hybrid Mouse Diversity Panel (HMDP) [21]. Those in linkage disequilibrium are represented by a single SNP with lowest P-value and nearest to the associated gene represents. Distance is the position of SNP site relative to the start position of the gene. For each SNP, expression levels of the associated genes in the aorta and macrophages are shown as ratios of two strains. Genes that show DBA-specific expression are bolded. ap<0.05, bp<0.01, cp<0.001. (DOC) [file pone.0088274.s010.doc]

**Table S4. SNPs within the *Ath44*** interval associated with gene expressions in the aorta.

| SNP | | Associated Gene | | | | | Aorta expression | | | Macrophage expression | | |
| --- | --- | --- | --- | --- | --- | --- | --- | --- | --- | --- | --- | --- |
| Position (Mb) | Name | Name | Chr | Position (Mb) | Distance (Mb) | P-value | DBA/129 | B6/129 | Level | DBA/129 | B6/129 | Level |
| 154.4 | rs31364674 | Rgl1 | 1 | 154.4 | +0.22 | 1.13E-19 | 1.09 | 0.91 | 162 | 1.13 | 2.82 | 332 |
| 155.0 | rs31170294 | Sox13 | 1 | 135.3 | -19.7 | 2.75E-09 | 0.96 | 1.10 | 154 | 0.98 | 0.70 | 51 |
| 155.0 | rs31170294 | Mrpl9 | 3 | 94.2 | trans | 2.05E-09 | 0.94 | 0.96 | 323 | 1.30 | 1.01 | 244 |
| 155.5 | rs30806276 | **Npl** | **1** | 155.4 | +0.06 | 6.90E-23 | 4.77^c^ | 1.36 | 40 | 5.88 | 0.64 | 289 |
| 156.6 | rs32397456 | Rnasel | 14 | 155.6 | trans | 1.23E-11 | 1.06 | 0.79 | 9 | 1.01 | 0.95 | 7 |
| 156.9 | rs31598960 | Ier5 | 1 | 156.9 | 0 | 4.63E-11 | 0.94 | 0.82 | 135 | 1.00 | 1.68 | 217 |
| 158.1 | rs31529590 | **Ifi202b** | **1** | 175.9 | -17.8 | 3.49E-08 | 1.66^b^ | 0.42^b^ | 16 | 1.62 | 0.03 | 238 |
| 158.4 | rs6380287 | **Tor3a** | 1 | 158.6 | +0.23 | 2.57E-20 | 1.53^a^ | 1.36 | 169 | 1.48 | 1.33 | 751 |
| 158.6 | rs13476194 | **Fam20b** | 1 | 158.6 | +0.07 | 6.16E-30 | 1.21^a^ | 0.90 | 692 | 1.42 | 0.80 | 753 |
| 163.3 | rs6282961 | **Fmo1** | 1 | 164.8 | +4.12 | 5.67E-08 | 0.41^b^ | 0.79 | 1850 | 0.92 | 1.00 | 37 |
| 163.5 | rs31446717 | Ifnab | 4 | 88.3 | trans | 2.84E-08 | 0.90 | 0.87 | 7 | 1.02 | 0.85 | 7 |
| 164.1 | rs32041876 | Ephx1 | 1 | 182.9 | +18.8 | 5.46E-08 | 1.01 | 1.04 | 1243 | 1.32 | 1.61 | 240 |
| 164.1 | rs32041876 | Agfg1 | 1 | 82.8 | +81.3 | 1.61E-07 | 0.95 | 0.96 | 2019 | 0.97 | 1.03 | 2235 |
| 164.1 | rs32041876 | Metap1 | 3 | 138.1 | trans | 2.13E-07 | 0.94 | 0.98 | 765 | 1.12 | 0.55 | 1539 |
| 164.1 | rs32041876 | Pitpnb | 5 | 111.8 | trans | 2.78E-07 | 0.91^a^ | 0.90^a^ | 1095 | 1.13 | 0.93 | 670 |
| 164.1 | rs32041876 | Tmed2 | 5 | 125.0 | trans | 1.55E-07 | 0.90^a^ | 0.92^a^ | 1801 | 1.01 | 0.97 | 1660 |
| 164.1 | rs32041876 | Tgoln1 | 6 | 72.6 | trans | 5.61E-07 | 0.90 | 0.98 | 1274 | 1.04 | 1.13 | 1087 |
| 164.1 | rs32041876 | Ptplad1 | 9 | 64.8 | trans | 5.23E-07 | 0.90 | 0.88 | 422 | 1.25 | 0.96 | 474 |
| 164.1 | rs32041876 | Tmem158 | 9 | 123.2 | trans | 5.99E-07 | 1.16 | 0.96 | 120 | 0.94 | 1.29 | 59 |
| 164.1 | rs32041876 | Krr1 | 10 | 111.4 | trans | 9.33E-07 | 0.98 | 0.92 | 408 | 1.06 | 1.53 | 1073 |
| 164.1 | rs32041876 | Kat2b | 17 | 53.7 | trans | 6.53E-07 | 1.01 | 0.96 | 538 | 1.09 | 1.13 | 294 |
| 164.1 | rs32041876 | Tbl1x | X | 74.8 | trans | 3.26E-07 | 1.12 | 1.13 | 1352 | 1.20 | 0.83 | 664 |
| 164.5 | rs32632664 | **Fmo2** | 1 | 164.8 | +0.11 | 3.38E-18 | 0.49^c^ | 0.90 | 4898 | 1.13 | 0.69 | 21 |
| 165.1 | rs6302874 | **Fmo3** | 1 | 164.9 | -0.18 | 5.28E-11 | 0.10^c^ | 0.64^b^ | 3870 | 1.36 | 1.09 | 17 |
| 165.9 | rs32552140 | Itm2b | 14 | 73.8 | trans | 2.20E-07 | 1.02 | 1.11^b^ | 4088 | 1.08 | 1.26 | 3913 |
| 166.3 | rs6404047 | **Creg1** | 1 | 167.7 | -1.42 | 5.27E-11 | 0.75^a^ | 0.92 | 1626 | 0.91 | 1.16 | 4237 |
| 166.9 | rs32650088 | Slc19a2 | 1 | 166.2 | +0.72 | 3.05E-15 | 0.84 | 0.97 | 121 | 1.30 | 0.94 | 142 |
| 167.0 | rs31737858 | Senp3 | 11 | 69.5 | trans | 2.01E-06 | 1.04 | 0.99 | 426 | 1.04 | 1.02 | 540 |
| 167.0 | rs31420643 | Itgb1 | 8 | 131.2 | trans | 1.31E-07 | 1.10 | 0.98 | 4055 | 1.09 | 0.83 | 2700 |
| 167.6 | rs31407749 | Selp | 1 | 166.0 | +1.53 | 5.48E-15 | 0.40 | 1.28 | 224 | 0.34 | 0.14 | 712 |
| 168.4 | rs33860546 | Clns1a | 7 | 104.8 | trans | 7.86E-07 | 1.06 | 1.04 | 11 | 0.97 | 1.31 | 11 |

SNPs within the 152.9 - 168.5 Mb interval of Chr 1, having nucleotide sharing pattern of 129 = B6 ≠ DBA and associated with genes at *P* < 1.00E-06 were selected from the eQTL data of the Hybrid Mouse Diversity Panel (HMDP) [21]. Those in linkage disequilibrium are represented by a single SNP with lowest *P*-value and nearest to the associated gene represents. Distance is the position of SNP site relative to the start position of the gene. For each SNP, expression levels of the associated genes in the aorta and macrophages are shown as ratios of two strains. Genes that show DBA-specific expression were bolded. ^a^p<0.05, ^b^p<0.01, ^c^p<0.001.
