# Supplementary material for: Quantitative Trait Loci Affecting Atherosclerosis at the Aortic Root Identified in an Intercross between DBA2J and 129S6 Apolipoprotein E-Null Mice
Source: PLoS One. 2014 Feb 20;9(2):e88274. doi: 10.1371/journal.pone.0088274 (PMC3930552; doi:10.1371/journal.pone.0088274)
Supplement: Table S5 — SNPs within the Ath45 interval associated with gene expressions in the aorta. SNPs within the 156.5 - 165.1 Mb interval of Chr 2, having nucleotide sharing pattern of 129 ≠ B6 = DBA, and associate with genes at P<1.00E-06 were selected from the eQTL data of the Hybrid Mouse Diversity Panel (HMDP) [21]. A SNP with lowest P-value and nearest to the associated gene represents those in linkage disequilibrium. Distance is the position of SNP site relative to the start position of the gene. For each SNP, expression levels of the associated genes in the aorta and macrophages are shown as ratios of two strains. ap<0.05, bp<0.01, cp<0.001. Genes that show 129-specific expression are bolded. (DOC) [file pone.0088274.s011.doc]

**Table S5. SNPs within the *Ath45* interval associated with gene expressions in the aorta.**

| SNP | | Associated Gene | | | | | Aorta expression | | | Macrophage expression | | |
| --- | --- | --- | --- | --- | --- | --- | --- | --- | --- | --- | --- | --- |
| Position (Mb) | Name | Name | Chr | Position (Mb) | Distance (Mb) | P-value | DBA/129 | B6/129 | Level | DBA/129 | B6/129 | Level |
| 155.7 | rs33679578 | **Dynlrb1** | 2 | 155.1 | -1.17 | 1.52E-11 | 1.44^b^ | 1.35^b^ | 1920 | 1.25 | 1.03 | 1915 |
| 155.8 | rs27325049 | Phf20 | 2 | 156.0 | +0.29 | 2.38E-23 | 0.74^a^ | 0.81 | 295 | 0.90 | 1.18 | 298 |
| 155.9 | rs3655350 | **Plac9** | 14 | 26.7 | trans | 1.01E-08 | 1.76^c^ | 1.74^a^ | 1107 | 1.05 | 1.10 | 34 |
| 157.5 | rs29919476 | Exosc1 | 19 | 42.0 | trans | 3.68E-07 | 1.00 | 0.86^a^ | 1038 | 1.01 | 0.84 | 1198 |
| 157.5 | rs27323422 | Ctnnbl1 | 2 | 157.6 | +0.07 | 5.99E-08 | 0.97 | 0.91 | 331 | 1.01 | 1.06 | 446 |
| 165.0 | rs33437128 | Sdc4 | 2 | 164.2 | -0.75 | 1.76E-07 | 0.86 | 0.94 | 1712 | 1.09 | 0.65 | 501 |

SNPs within the 156.5 - 165.1 Mb interval of Chr 2, having nucleotide sharing pattern of 129 ≠ B6 = DBA, and associate with genes at *P* < 1.00E-06 were selected from the eQTL data of the Hybrid Mouse Diversity Panel (HMDP) [21]. A SNP with lowest *P*-value and nearest to the associated gene represents those in linkage disequilibrium. Distance is the position of SNP site relative to the start position of the gene. For each SNP, expression levels of the associated genes in the aorta and macrophages are shown as ratios of two strains. ^a^p<0.05, ^b^p<0.01, ^c^p<0.001. Genes that show 129-specific expression are bolded.
